# SPARC Drives Tubulointerstitial Fibrosis through Regulating the CBP-DOT1L Pathway

**DOI:** 10.7150/ijbs.133879

**Published:** 2026-05-18

**Authors:** Huimin Jiang, Qing Yang, Kuo Wang, Qinqin Tan, Chenyang Xiong, Xindi Zhou, Chun Gan, Han Xiao, Lan Qiu, Yaxi Chen, Mo Wang, Haiping Yang, Wei Jiang, Qiu Li

**Affiliations:** 1 Department of Nephrology, Children's Hospital of Chongqing Medical University, National Clinical Research Center for Children and Adolescents' Health and Diseases, Ministry of Education Key Laboratory of Child Development and Disorders, Chongqing Key Laboratory of Pediatric Metabolism and Inflammatory Diseases, Chongqing, P.R. China.; 2 Centre for Lipid Research & Key Laboratory of Molecular Biology for Infectious Diseases (Ministry of Education), Institute for Viral Hepatitis, Department of Infectious Diseases, the Second Affiliated Hospital of Chongqing Medical University, Chongqing, P.R. China.

**Keywords:** SPARC, tubulointerstitial fibrosis, CKD, PTECs, DOT1L, CBP

## Abstract

Renal tubulointerstitial fibrosis (TIF) is a central pathological feature driving the progression of chronic kidney disease (CKD) toward end-stage renal failure. Despite advances in understanding fibrotic mechanisms, effective anti-fibrotic therapies remain limited. Here, we identify SPARC, a matricellular protein expressed in proximal tubular epithelial cells (PTECs), as a key mediator of TIF. SPARC expression strongly correlates with fibrosis severity in both human CKD biopsies and murine models of unilateral ureteral obstruction (UUO) and ischemia-reperfusion injury (IRI). Genetic ablation of Sparc markedly attenuates renal fibrosis in these models. Mechanistically, SPARC stabilizes DOT1L protein, enhancing H3K79 di-methylation (H3K79me2) and promoting fibrotic changes in PTECs. This process is orchestrated by the acetyltransferase CBP, whose regulation of DOT1L stability depends on MEK-ERK signaling. The SPARC-CBP-DOT1L axis thus defines a previously unrecognized epigenetic pathway driving renal fibrosis. Our findings establish SPARC as a critical driver of TIF and highlight the SPARC-CBP-DOT1L signaling cascade as a promising therapeutic target for halting fibrotic progression in CKD.

## Introduction

Renal tubulointerstitial fibrosis (TIF) is a critical pathological process in the progression of chronic kidney diseases (CKD) and serves as the final common pathway to end-stage renal disease (ESRD)^1^-^3^. It is characterized by the excessive accumulation of extracellular matrix (ECM) in the renal interstitium, which can be observed on kidney biopsy. The extent of TIF serves as an important prognostic marker and guides treatment decisions. This process involves fibroblast activation, persistent inflammation, and ECM accumulation, ultimately leading to replacement of functional parenchyma with scar tissue and loss of renal function^4^-^8^. Despite significant advances in understanding the mechanisms of renal fibrosis, its pathogenesis remains complex, involving multiple, overlapping cellular and molecular pathways. TIF stands as the central pathological event in this process, driving the progression of kidney injury^8^. Thus, a deeper understanding of TIF is essential for developing novel therapeutic strategies to prevent or halt the progression of CKD.

The initiation of TIF is driven by diverse factors, including chronic kidney injury, dysregulated immune responses, hemodynamic stress, and metabolic disturbances^6^. Proximal tubular epithelial cells (PTECs) have emerged as central mediators of tubulointerstitial fibrosis. After injury stimulation, PTECs undergo phenotypic and metabolic alterations that directly contribute to fibrogenesis. Activated PTECs also function as signaling hubs, secreting profibrotic mediators such as transforming growth factor-β1 (TGF-β1), a pleiotropic cytokine and master regulator of fibrosis^7^-^10^. TGF-β1 promotes fibrogenesis through both canonical (Smad-dependent) and non-canonical (Smad-independent) signaling pathways, leading to myofibroblast activation, excessive ECM production, and impaired ECM degradation^11^-^13^. The role of Smad proteins in fibrosis regulation is multifaceted, displaying both profibrotic and antifibrotic effects, particularly in the context of mesenchymal transition. Moreover, the intricate interplay between TGF-β/Smad signaling and other molecular pathways further complicates the pathogenesis of TIF. While fibrosis can also be triggered by factors beyond inflammation, such as mechanical stress, metabolic changes, or direct cellular injury, PTECs injury in TIF can contribute to fibrosis even in the absence of overt inflammation^14^,^15^. These considerations underscore the need to identify additional molecular regulators of renal fibrosis.

The secreted protein acidic and rich in cysteine (SPARC), also known as BM-40 and osteonectine, is a multifunctional glycoprotein within the matricellular protein family^16^. SPARC could modulate cellular interactions with the ECM by binding structural matrix proteins, influencing ECM remodeling, and regulating cellular behavior^17^,^18^. Notably, SPARC has been implicated in a variety of physiological and pathological contexts, including tissue injury and wound healing, making it a compelling candidate for investigation in renal fibrogenesis^19^,^20^. Additionally, the disruptor of telomeric silencing-1 like (DOT1L) protein catalyzes histone H3 methylation at Lys79 (H3K79), playing a key role in transcriptional regulation, cell cycle control, DNA repair, and various developmental processes^21^,^22^. Targeting DOT1L has been shown to attenuate renal fibrosis by inhibiting renal fibroblasts and epithelial-mesenchymal transition (EMT) 23. In this study, we have identified the SPARC as a novel mediator of TIF. We show that SPARC promotes fibrosis through a specific molecular axis in which it regulates CBP via the MEK-ERK signaling, enhancing DOT1L protein stability and increasing H3K79me2, a critical epigenetic mark in fibrogenesis. By exploring the SPARC-CBP-DOT1L pathway, our findings provide new insights into the mechanisms underlying TIF, suggesting that SPARC may serve as a promising target for therapeutic strategies aimed at alleviating renal fibrosis.

## Materials and Methods

### Human kidney samples

Renal biopsy specimens were obtained from the Department of Nephrology, Children's Hospital Affiliated to Chongqing Medical University. Normal control samples were collected from peritumoral tissues of patients undergoing nephrectomy for renal tumors. The basic information of the patients is summarized in Supplementary Table 1. The study protocol was approved by the Research Ethics Committee of the Children's Hospital of Chongqing Medical University (Ethics Approval No. 2025442). This study enrolled patients with diabetic nephropathy at the Affiliated Hospital of North Sichuan Medical College, with approval from its Ethics Committee (File Number: 2025ER443-1). Renal interstitial fibrosis was assessed according to the Banff classification using Masson's trichrome staining. The fibrosis area was quantified relative to the total renal cortex area. The fibrosis was graded as follows: Grade 0 (no fibrosis, <5%), Grade 1 (mild fibrosis, 5%-25%), Grade 2 (moderate fibrosis, 25%-50%), and Grade 3 (severe fibrosis, >50%).

### Renal function assessment

Blood urea nitrogen (BUN), serum Creatinine (Scr), and serum uric acid levels were measured using commercially available kits (C013-2-1, C011-2-1, C012-1-1; Nanjing Jiancheng Bioengineering Institute, China) according to the manufacturer's instructions.

### LC-MS/MS proteomics

After treatment, HK-2 cells were digested with trypsin, washed, and frozen at -80°C. Quantitative proteomic analysis was performed using 4D-FastDIA, and differential proteins were identified based on fold change and significance *P* values from T-test analysis. The Fuzzy C-Means Clustering (FCM) algorithm, implemented via the R package TCseq (version 1.8.0), was used to conduct clustering analysis on the expression patterns of protein abundance changes across different groups of continuous samples.

### Co-immunoprecipitation (Co-IP)

HA-CBP and Flag-DOT1L plasmids were co-transfected into HK-2 cells using Lipofectamine 3000 (L3000075, Invitrogen, USA). After 48 h, cell lysates were pre-cleared with Protein A/G magnetic beads (B23202, Selleck, USA), then incubated with anti-HA, anti-Flag, or isotype IgG antibodies at 4°C for 2 h. Immunoprecipitates were analyzed by Western blot. For endogenous co-IP, HK-2 cells were lysed and incubated overnight at 4°C with anti-DOT1L (ab72454, Abcam, USA) or anti-acetyl-lysine (ab190479, Abcam, USA) antibodies. For detecting SPARC-Integrin β1 interaction on the cell membrane, cells were lysed with a mild lysis buffer to maintain membrane integrity, followed by co-IP using anti-SPARC (ab207743, Abcam, USA) or anti-Integrin β1 (ab183666, Abcam, USA) antibodies. Complexes were captured with Protein A/G beads, washed, and immunoblotted to detect protein-protein interactions.

### Statistical analyses

Data were analyzed using GraphPad Prism 10. Results are presented as mean ± SD. Two-group comparisons were performed with a two-tailed Student's t-test. For multiple comparisons, one-way ANOVA followed by Tukey's test was used. Pearson's correlation and linear regression were applied for correlation analysis. A *P* value < 0.05 was considered statistically significant.

## Results

### The elevated SPARC expression is associated with increased severity of TIF

**Figure 1:**
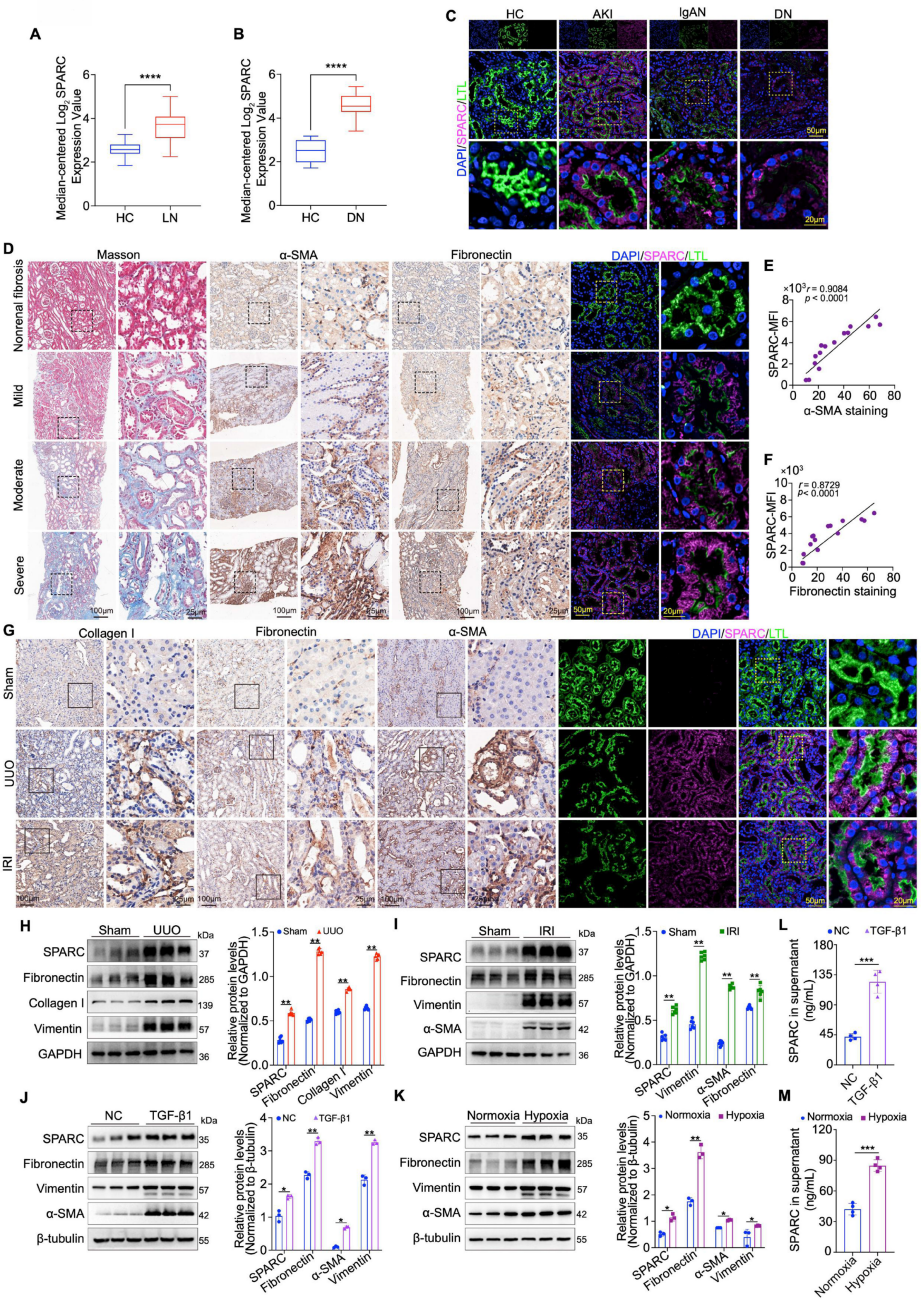
** SPARC expression correlates with TIF progression in humans and mice**. **A** and** B** Relative *SPARC* mRNA levels in the renal tissue, analyzed using data from the Nephroseq database (http://www.nephroseq.org). **C** Representative images showing colocalization of SPARC and proximal tubules marker LTL in kidney tissues from patients with AKI, IgAN and DN. Non-tumor kidney tissues from patients with renal carcinoma served as healthy controls (HC). **D** Images of Masson staining, immunohistochemical staining of α-SMA and Fibronectin, and immunofluorescence staining of SPARC in patients with fibrosis. **E** and** F** Pearson's correlation analysis showing the relationship between mean fluorescence intensity of SPARC and immunohistochemical staining of α-SMA or Fibronectin in patients with fibrosis (n=15). **G** Representative images of immunohistochemical staining for Collagen I, Fibronectin, and α-SMA, and immunofluorescence showing colocalization of SPARC with the proximal tubule marker LTL in UUO and IRI mouse kidneys. **H** and** I** Western blot and quantitative data analysis of SPARC, Fibronectin, Collagen I, Vimentin and α-SMA in kidneys of UUO and IRI mouse groups (n=6). **J** and **K** Western blot analysis of SPARC, Fibronectin, Vimentin and α-SMA in HK-2 cells treated with 10ng/ml TGF-β1 or 700 μM CoCl_2_ (n=3). **L** and **M** ELISA analysis of SPARC secretion in HK-2 cells treated with TGF-β1 or CoCl_2_. Data are presented as the mean ± SD. Statistical significance is indicated as *ns* (not significant), *P < 0.05, **P < 0.01, ***P < 0.001.

To investigate the relationship between SPARC expression and the severity of TIF, we first utilized the Nephroseq database, a comprehensive platform for kidney disease research and gene expression analysis. We assessed renal SPARC expression in lupus nephritis (LN) and diabetic nephropathy (DN), observing significantly elevated levels in both conditions (Fig. 1A, B). Building on this, we performed immunofluorescence staining to examine SPARC expression in renal tubules across a clinical cohort of renal biopsy specimens, including samples from patients with acute kidney injury (AKI), IgA nephropathy (IgAN), and DN. These analyses consistently revealed increased SPARC expression in all three conditions, with the expression primarily localized to PTECs, as marked by LTL co-staining (Fig. 1C). To assess whether elevated SPARC expression correlates with TIF severity, we performed immunohistochemical staining on biopsy specimens with varying fibrosis degrees. We assessed the expression of fibrosis markers α-SMA and Fibronectin, along with dual immunofluorescence labeling for LTL and SPARC, followed by correlation analysis (Fig. 1D-F). The results showed a positive correlation between SPARC expression and both α-SMA and Fibronectin levels. To further investigate the role of SPARC in TIF, we established two mouse models of renal fibrosis, unilateral ureteral obstruction (UUO) and ischemia-reperfusion injury (IRI). Biochemical analysis revealed significant renal dysfunction in both UUO and IRI mice (Fig. S1A, B). Light microscopy findings confirmed tubular vacuolation, as shown in hematoxylin and eosin (HE) staining and periodic acid-Schiff (PAS) staining, and substantial fibrosis, as demonstrated in Masson trichrome and Sirius red staining (Fig. S1C, D). We then re-evaluated the expression of the fibrosis marker proteins α-SMA, Fibronectin and Collagen I via immunohistochemical staining, combined with dual immunofluorescence labeling for LTL and SPARC. The results reaffirmed the findings from the previous clinical kidney biopsy findings, showing a positive correlation between SPARC expression and both α-SMA and Fibronectin expression (Fig. 1G). Additionally, we detected the expression of SPARC and fibrosis-related proteins in mouse kidney tissues via Western blot analysis. The results showed that SPARC expression consistently increased alongside fibrosis-related proteins detected in UUO (Fibronectin, Collagen I, Vimentin) and IRI (Fibronectin, Vimentin, α-SMA) (Fig. 1H, I). *In vitro* experiments with HK-2 cells showed a significant increase in SPARC expression under TGF-β1-induced inflammatory conditions and cobalt dichloride (CoCl_2_)-induced hypoxic conditions (Fig. S1E, F). This increase in SPARC was associated with elevated levels of Fibronectin, Vimentin, and α-SMA (Fig. 1J, K). Analysis of culture supernatants showed that SPARC secretion was also markedly elevated under both TGF-β1 and CoCl_2_ treatment (Fig. 1L, M). Together, these findings demonstrate a consistent association between elevated SPARC expression and the increased severity of TIF.

### The ablation of SPARC significantly attenuates TIF

**Figure 2:**
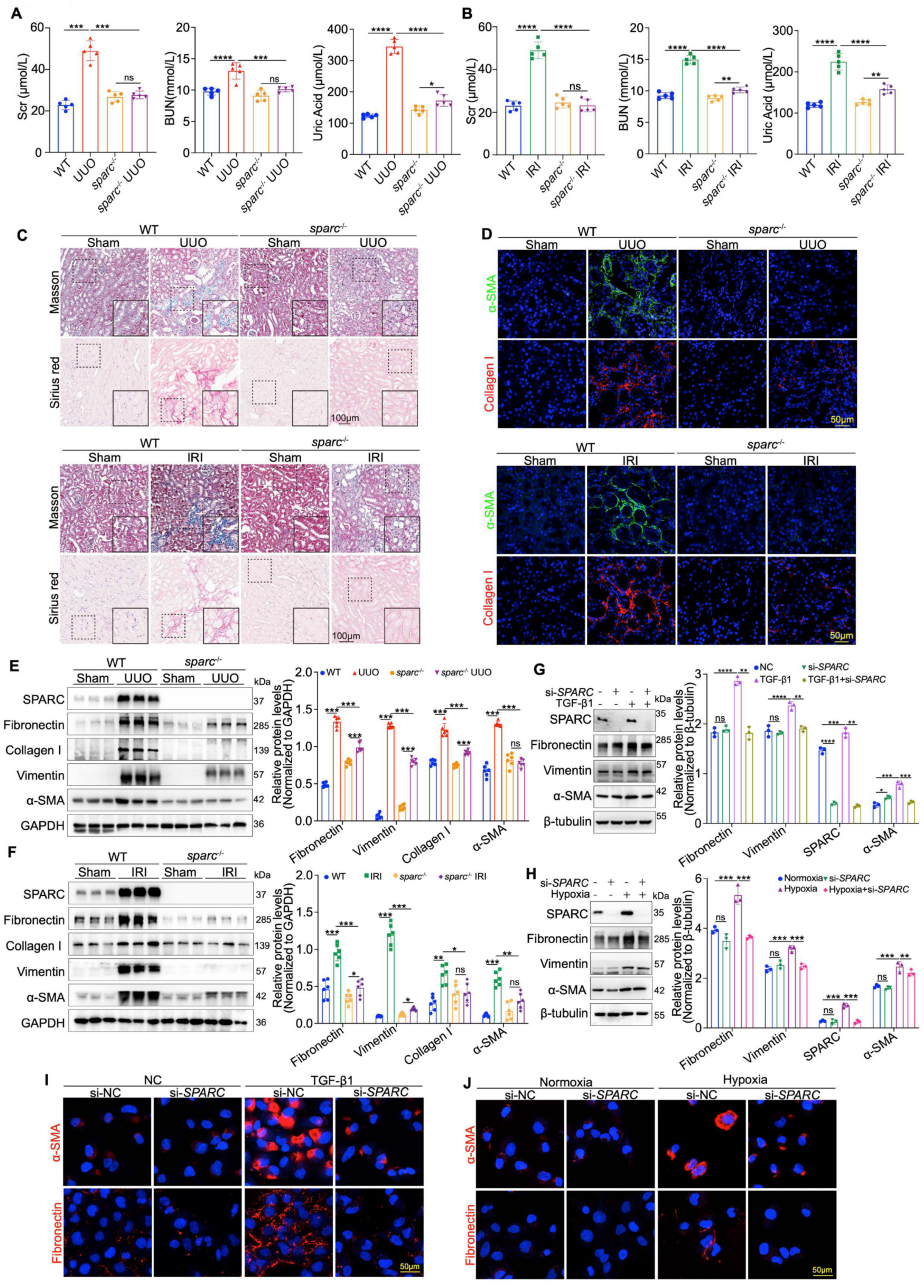
** Ablation of SPARC significantly attenuates tubulointerstitial fibrosis (TIF). A** and** B** Serum creatinine (Scr), blood urea nitrogen (BUN), and uric acid levels in WT and *sparc*⁻/⁻ mice following UUO and IRI surgery. **C** Representative images of Masson and Sirius red staining in kidney sections from WT and *sparc*^-/-^ mice after UUO and IRI. **D** Confocal microscopy staining of α-SMA and Collagen I in the kidney sections of WT and *sparc*^-/-^ mice following UUO and IRI. **E** and **F** Western blot and quantitative data showing the expression of SPARC, Fibronectin, Collagen I, Vimentin and α-SMA in the kidneys of WT and *sparc*^-/-^ mice after UUO and IRI (n=6). **G** and** H** Western blot and quantitative data showing the expression of SPARC, Fibronectin, Vimentin and α-SMA in HK-2 cells which treated with TGF-β1 or CoCl_2_ following *SPARC* knockdown (n=3). **I** and** J** Confocal microscopy staining of α-SMA and Fibronectin in HK-2 cells which treated with TGF-β1 or CoCl_2_ after *SPARC* knockdown. Data are presented as the mean ± SD. Statistical significance is indicated as *ns* (not significant), *P < 0.05, **P < 0.01, ***P < 0.001.

Having established the correlation between elevated SPARC expression and TIF severity, we next sought to determine if SPARC ablation could reduce TIF severity. To this end, *SPARC* knockout (*sparc*^-/-^) mice were generated and subsequently subjected to UUO and IRI-induced renal fibrosis (Fig. S2A, B). Biochemical analysis revealed that SPARC ablation significantly improved elevated BUN, Scr, and uric acid levels in both UUO and IRI mice (Fig. 2A, B). Light microscopy findings further demonstrated that *SPARC* knockout significantly improved tubular vacuolation, as shown by HE and PAS staining, and reduced fibrosis, as evidenced by Masson and Sirius red staining. Additionally, Fibronectin expression was significantly decreased, as confirmed by immunohistochemical analysis (Fig. 2C and Fig. S2C, D). Further immunofluorescence staining confirmed that SPARC ablation significantly reduced the expression of fibrotic proteins Collagen I and α-SMA (Fig. 2D). Western blot analysis consistently showed that *SPARC* knockout suppressed the expression of fibrosis-related proteins, including Fibronectin, Collagen I, Vimentin, and α-SMA in both UUO and IRI models (Fig. 2E, F). *In vitro* experiments with HK-2 cells showed that interference with SPARC expression suppressed the TGF-β1-induced increase in Fibronectin, Vimentin, and α-SMA levels, as well as the CoCl_2_-induced increase in these proteins under hypoxic conditions. These findings were further confirmed by immunofluorescence analysis (Fig. 2G-J). In contrast, incubation of HK-2 cells with recombinant SPARC protein exacerbated the expression of these fibrotic markers (Fig. S2E-G). Together, these results suggest that SPARC ablation effectively attenuates TIF.

### DOT1L mediate SPARC-driven regulation of TIF in PTECs

**Figure 3:**
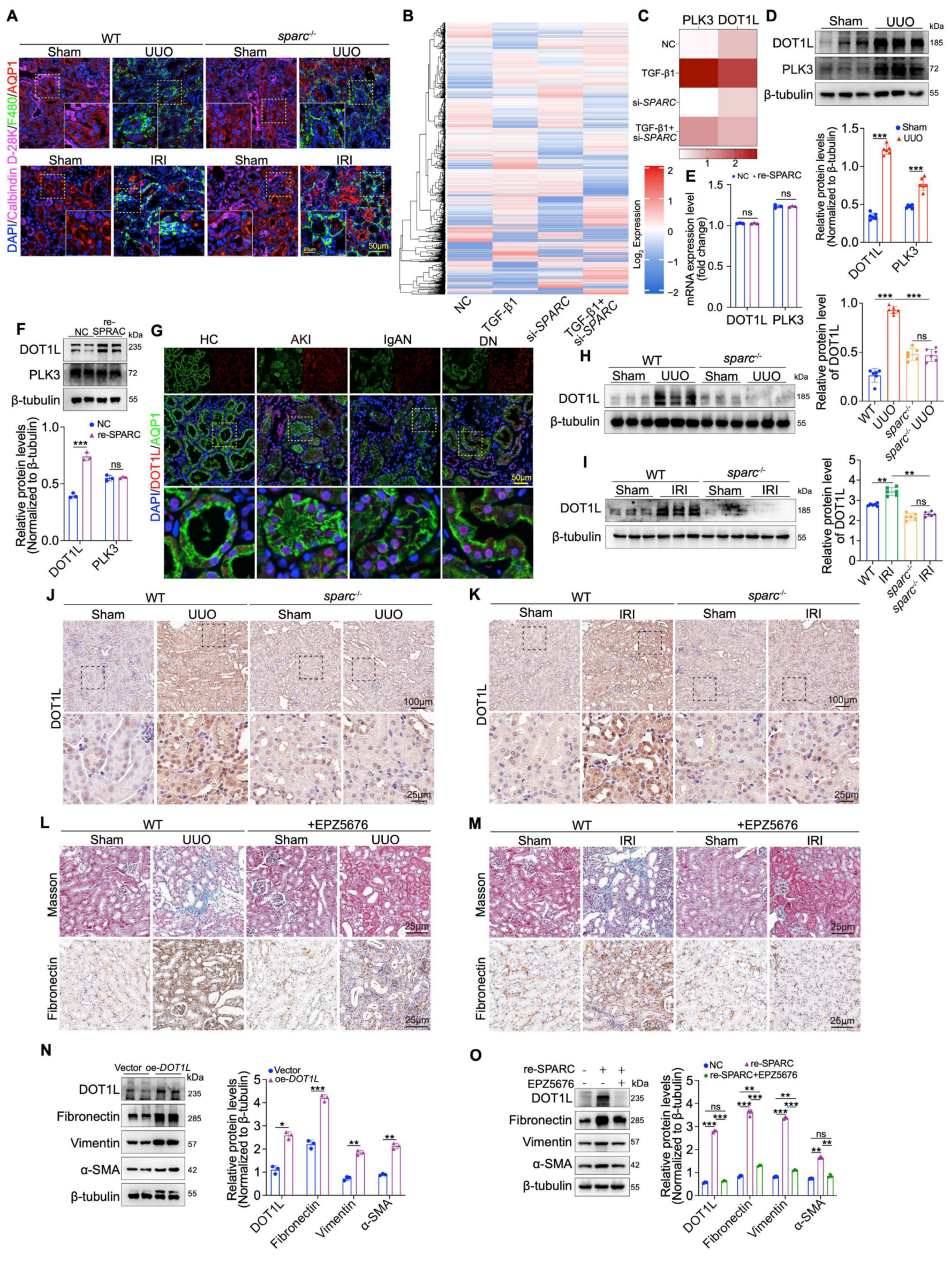
** SPARC regulates DOT1L to participate in tubulointerstitial fibrosis**. **A** Immunoflu- orescence staining showing colocalization of macrophage marker protein F4/80 with AQP1 and Calbindin-D28k in the kidney sections from WT and *sparc*^-/-^ mice after UUO and IRI. **B** Heatmap of differentially expressed protein among HK-2 cells across different treatment groups. **C** Heatmap of the expression levels of PLK3 and DOT1L in the indicated groups. **D** Western blot and quantitative data showing the expression of DOT1L and PLK3 from UUO mice kidneys (n=6). **E** Relative mRNA expression of *DOT1L* and *PLK3* in NC and SPARC-treatment groups determined by qPCR (n=3). **F** Western blot showing DOT1L and PLK3 expression in HK-2 cells incubated with 0.5 μg/ml recombinant SPARC protein for 48h (n=3). **G** Immunofluorescence images showing colocalization of DOT1L and AQP1 in kidney tissues from patients with AKI, IgAN, and DN. **H** and** I** Western blot and quantitative data showing DOT1L protein expression in kidneys of WT and *sparc*^-/-^ mice after UUO and IRI. **J** and** K** Immunohistochemical staining of DOT1L in kidneys of WT and *sparc*^-/-^ mouse kidneys after UUO and IRI. **L** and** M** Representative images of renal Masson, and Fibronectin staining of WT mice injected or not injected with the DOT1L inhibitor (EPZ5676) after UUO and IRI. **N** Western blot and quantitative data showing the expression of DOT1L, Fibronectin, Vimentin and α-SMA in HK-2 cells overexpressing DOT1L (n=3). **O** Western blot and quantitative data showing the expression of DOT1L, Fibronectin, Vimentin and α-SMA in HK-2 cells pretreated with the EPZ5676 prior to incubation with recombinant SPARC protein (n=3). Data are presented as the mean ± SD. Statistical significance is indicated as *ns*, no significance, *P < 0.05, **P < 0.01, ***P < 0.001.

Given the critical role of renal macrophage infiltration in TIF development, we sought to determine whether SPARC regulates macrophage infiltration by acting on the PTECs where it is expressed^24^,^25^. To address this, we investigated the effect of *SPARC* knockout on macrophage infiltration in kidneys subjected to IRI and UUO. Triple immunofluorescence staining, labeling macrophages (F480), proximal tubules (AQP1), and distal tubules (Calbindin D-28K), appeared to be no significant difference in macrophage infiltration between *sparc*^-/-^ and wild-type (WT) IRI and UUO kidneys (Fig. 3A), suggesting that macrophages are not the sole mediators of SPARC-induced fibrosis. Consequently, we explored the underlying fibrosis regulatory mechanism by disrupting SPARC expression in TGF-β1-stimulated PTEC cells using tandem mass spectrometry analysis. Through protein expression heatmap analysis, quantitative assessment of up- and downregulated proteins, and a comprehensive analysis of protein clusters, we identified DOT1L and Polo-Like Kinase 3 (PLK3) as key proteins involved in this process (Fig. 3B, C and Fig. S3A, B). We then examined the expression of these proteins in kidney tissue from UUO mouse models via Western blot, which confirmed their elevated expression under pathological conditions (Fig. 3D). To further determine the mechanism of upregulation, HK-2 cells were incubated with recombinant SPARC protein. SPARC stimulation did not significantly alter *DOT1L* or *PLK3* mRNA levels (Fig. 3E). At the protein level, only DOT1L was markedly increased, whereas PLK3 remained unchanged (Fig. 3F). Further immunofluorescence staining of renal biopsy specimens revealed high DOT1L expression in the nuclei of pathological PTECs (Fig. 3G). *In vivo*, *SPARC* knockout significantly reduced DOT1L expression in both UUO and IRI models, as confirmed by Western blot and immunohistochemical analysis (Fig. 3H-K). These findings suggest that DOT1L may mediate SPARC-driven regulation of fibrosis. To validate the role of DOT1L in TIF, we used EPZ5676 (Pinometostat), a DOT1L inhibitor, to assess its effects on renal fibrosis in UUO and IRI mouse models. Administration of EPZ5676 significantly improved tubular vacuolation, as shown by HE and PAS staining, and reduced fibrosis, as evidenced by Masson and Sirius red staining (Fig. 3L, M and Fig. S3C, D). Immunohistochemical and Western blot analyses confirmed that EPZ5676 treatment significantly suppressed the expression of fibrosis markers, including Fibronectin, Collagen I, Vimentin, and α-SMA, in both UUO and IRI mouse kidney tissues (Fig. S3C-F). In contrast, overexpression of DOT1L in HK-2 cells increased fibrosis-related proteins (Fig. 3N). Interference with DOT1L effectively mitigated the TGF-β1- and CoCl_2_-induced increases in these proteins (Fig. S3G-J). Furthermore, treatment with EPZ5676 inhibited the expression of fibrosis-related proteins in HK-2 cells incubated with recombinant SPARC protein (Fig. 3O). These results suggest that DOT1L mediates SPARC-driven regulation of TIF in PTECs.

### SPARC promotes TIF via upregulation of DOT1L-mediated H3K79me2

**Figure 4:**
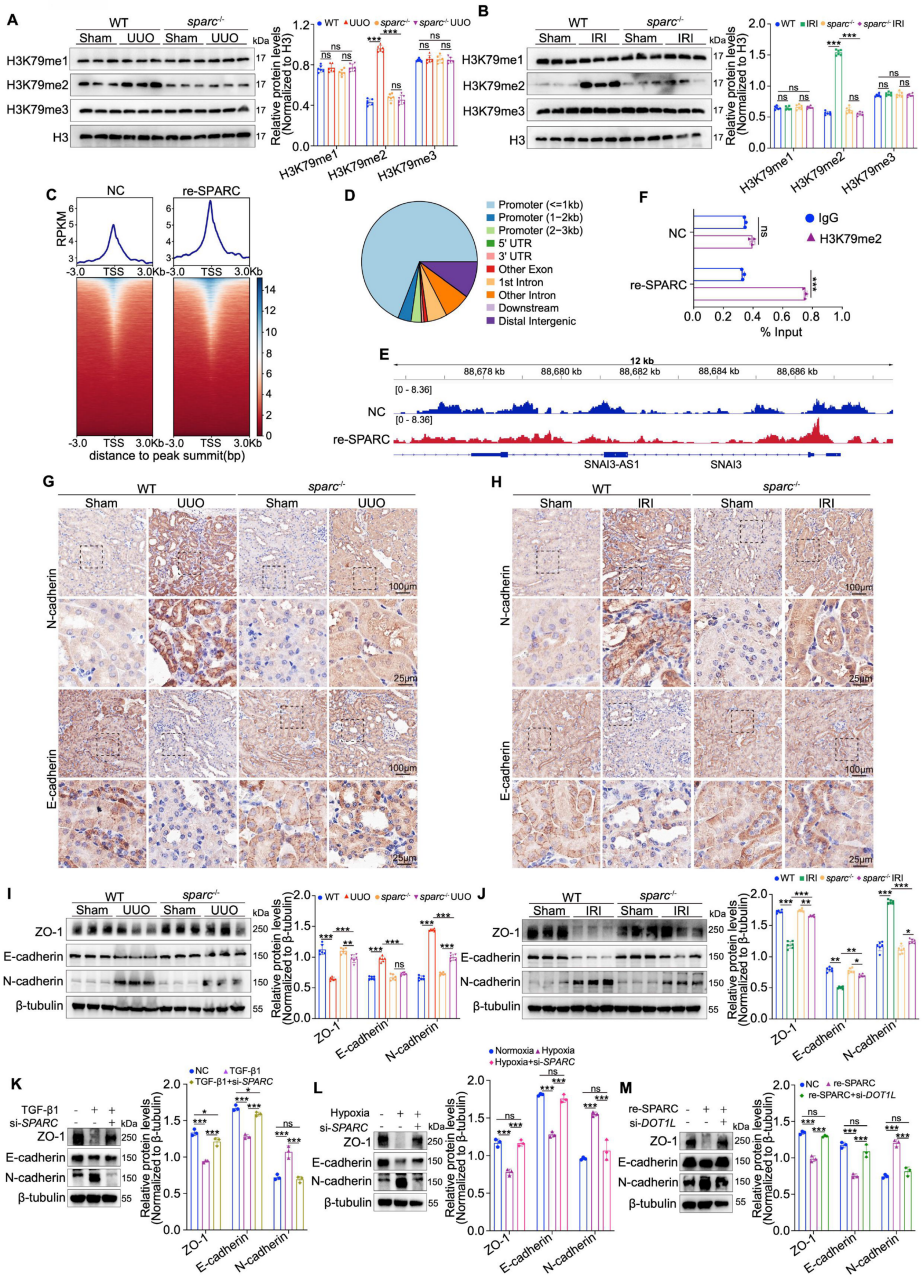
** SPARC promotes TIF by regulating DOT1L-mediated H3K79 di-methylation in PTECs. A** and** B** Western blot and quantitative data showing the expression of H3K79me1, H3K79me2 and H3K79me3 in the kidneys of WT and *sparc*^-/-^ mice after UUO and IRI. **C** Heatmaps of CUT&Tag signal intensity at H3K79me2 binding peaks in HK-2 cells, comparing the Control (NC) and SPARC-treatment (re-SPARC) groups. The color bar represents the normalized read density of the assay. **D** Genomic distribution of H3K79me2 binding peaks in HK-2 cells. **E** IGV tracks showing H3K79me2 enrichment at the *SNAI3* locus in NC and re-SPARC groups. **F** ChIP-qPCR analysis of H3K79me2 occupancy at the *SNAI3* promoter in NC and re-SPARC groups. **G** and** H** Immunohistochemical staining for E-cadherin and N-cadherin in kidney sections from WT and *sparc*^-/-^ mice after UUO and IRI. **I** and** J** Western blot and quantitative data showing the expression of ZO-1, E-cadherin and N-cadherin in the kidneys of WT and *sparc*^-/-^ mice after UUO and IRI (n=6). **K** and** L** Western blot analysis of expression of ZO-1, E-cadherin and N-cadherin in HK-2 cells which treated with TGF-β1 or CoCl_2_ following *SPARC* knockdown (n=3). **M** Western blot analysis expression of ZO-1, E-cadherin and N-cadherin in HK-2 cells which incubated with SPARC recombinant protein following *DOT1L* knockdown (n=3). Data are presented as the mean ± SD. Statistical significance is indicated as *ns*, no significance, *P < 0.05, **P < 0.01, ***P < 0.001.

DOT1L is a histone methyltransferase that catalyzes the addition of methyl groups to histone H3 at the lysine 79 residue, a modification crucial for regulating gene transcription^26^,^27^. To determine whether SPARC affects this modification in the context of TIF, we measured H3K79me1/2/3 levels in kidney tissues from UUO and IRI models using Western blot. We observed that only H3K79me2 levels were significantly reduced upon SPARC loss, while H3K79me1 and H3K79me3 remained unchanged (Fig. 4A, B). Further confirmation was obtained through immunofluorescence and immunohistochemical staining, which also demonstrated a reduction in H3K79me2 modification following SPARC ablation (Fig. S4A-D). To investigate SPARC's effect on H3K79me2 deposition at the genomic level, we performed CUT&Tag sequencing analysis in HK-2 cells treated with recombinant SPARC. This analysis revealed global enrichment of H3K79me2 at transcription start sites and confirmed that most H3K79me2 peaks localized to promoter regions (Fig. 4C, D). Notably, SPARC significantly increased H3K79me2 occupancy at the *SNAI3* locus, which was further validated by ChIP-qPCR (Fig. 4E, F). Consistently, qPCR results showed that SPARC significantly upregulated *SNAI3* mRNA expression, an effect that was reversed by DOT1L knockdown (Fig. S4E). These results indicate that SPARC promotes H3K79me2 modification at the *SNAI3* locus. *SNAI3* along with *SNAI1* and *SNAI2* belong to the snail family, encoding transcriptional repressors implicated in TIF^28^. Further, SPARC increased the expression of other key TIF transcription factors, including *Snai1*, *Snai2*, *Twist1* and *Zeb* (Fig. S4F, G). We then analyzed changes in E-cadherin and N-cadherin expression through immunohistochemistry and Western blot analysis, as the conversion from E-cadherin to N-cadherin is a hallmark of EMT. In UUO and IRI fibrotic mouse models, the reduced expression of ZO-1 and E-cadherin, and increased expression of N-cadherin in renal tissues were significantly suppressed following SPARC knockout (Fig. 4G-J). *In vitro* experiments with HK-2 cells showed that SPARC interference suppressed the TGF-β1-induced decrease in E-cadherin and ZO-1, and the increase in N-cadherin. These effects were also observed under hypoxic conditions induced by CoCl_2_ (Fig. 4K, L). Moreover, interfering with DOT1L expression suppressed SPARC-induced changes in these fibrosis markers (Fig. 4M). Taken together, these findings demonstrate that SPARC promotes H3K79me2 modification through upregulating DOT1L expression to drive TIF.

### CBP mediates SPARC-dependent maintenance of DOT1L protein stability

**Figure 5:**
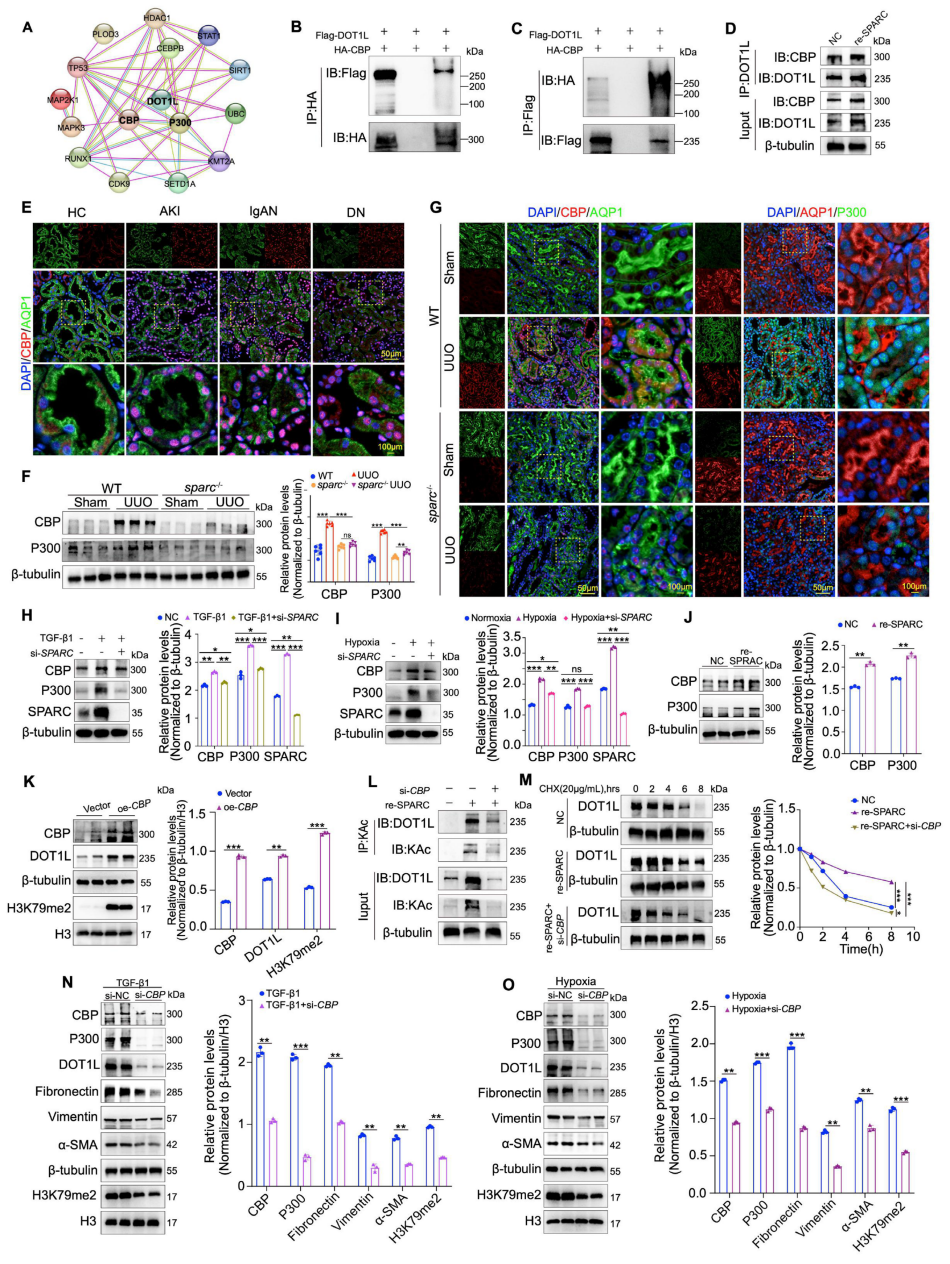
** CBP interacts with DOT1L and facilitates acetylation modification. A** Prediction of DOT1L-interacting proteins based on STRING database analysis. **B** and** C** 293T cells were co-transfected with HA-tagged CBP and Flag-tagged DOT1L expression plasmids. Cell lysates were immunoprecipitated with anti-HA or anti-Flag antibodies, followed by immunoblotting to detect CBP-DOT1L interactions. **D** Co-IP assays showing that incubation with recombinant SPARC protein significantly enhances the physical interaction between CBP and DOT1L in HK-2 cells. **E** Immunofluorescence images showing colocalization of CBP with the proximal tubular marker AQP1 in kidney tissues from patients with AKI, IgAN and DN. **F** Western blot and quantitative data showing the expression of CBP and P300 in the kidneys of WT and *sparc*^-/-^ mice after UUO (n=6). **G** Representative immunofluorescence images showing colocalization of CBP and P300 with AQP1 in kidney sections from WT and *sparc*^-/-^ mice after UUO. **H** and **I** Western blot and quantitative data showing the expression of CBP and P300 in HK-2 cells which treated with TGF-β1 or CoCl_2_ following *SPARC* knockdown (n=3). **J** Expression of CBP and P300 in HK-2 cells incubated with recombinant SPARC protein (n=3). **K** Western blot and quantitative analysis showing DOT1L and H3K79me2 expression in HK-2 cells transfected with CBP overexpression plasmid (n=3). **L** Immunoprecipitation with an anti-acetyl-lysine (KAc) antibody followed by immunoblotting for DOT1L in HK-2 cells incubated with recombinant SPARC incubation after CBP knockdown. **M** CHX chase assay showing that recombinant SPARC treatment significantly prolonged the half-life of DOT1L, while CBP knockdown abrogated this stabilizing effect. **N** and** O** Western blot and quantitative data showing protein expression in HK-2 cells which treated with TGF-β1 or CoCl_2_ following CBP knockdown (n=3). Data are presented as the mean ± SD. Statistical significance is indicated as *ns*, no significance, *P < 0.05, **P < 0.01, ***P < 0.001.

While DOT1L has been shown to mediate SPARC-driven regulation of TIF in PTECs, the molecular mechanism linking SPARC to DOT1L remains unclear, especially given that SPARC does not upregulate DOT1L mRNA levels. To explore this, we used the STRING (Functional Protein Interaction Network) database to identify molecules potentially associated with DOT1L. The analysis revealed that CBP and P300 may interact with DOT1L (Fig. 5A). CBP and P300 are lysine acetyltransferases that acetylate both histone and non-histone proteins to regulate gene expression and other cellular processes^29^. To validate this interaction, we performed immunoprecipitation assays, which demonstrated a direct protein interaction between CBP and DOT1L, which was enhanced upon SPARC overexpression (Fig. 5B-D). Analysis of clinical renal biopsy specimens showed that CBP is significantly overexpressed in the nuclei of PTECs (Fig. 5E). To further explore its relationship with SPARC, we examined whether SPARC knockout affected CBP and P300 expression in renal fibrosis models of UUO and IRI. Western blot and immunofluorescence analyses revealed that SPARC ablation significantly suppressed CBP and P300 expression in PTECs in both models (Fig. 5F, G and Fig. S5A-C). *In vitro,* SPARC knockdown prevented TGF-β1- and CoCl_2_-induced upregulation of CBP and P300, whereas recombinant SPARC treatment increased their expression in HK-2 cells (Fig. 5H-J). Overexpression of CBP in HK-2 cells resulted in increased DOT1L expression, accompanied by elevated fibrosis markers, including Fibronectin, Collagen I, Vimentin, and α-SMA, and increased H3K79me2 (Fig. 5K and Fig. S5D). Conversely, CBP knockdown significantly reduced DOT1L expression (Fig. S5E). Given that *DOT1L* mRNA levels remained unchanged, we investigated protein acetylation as a potential mechanism^30^. Acetylation immunoprecipitation assays demonstrated that SPARC overexpression enhanced DOT1L acetylation, which was reversed by CBP knockdown (Fig 5L). Cycloheximide chase experiments further showed that SPARC prolonged DOT1L protein half-life, whereas CBP knockdown attenuated this effect (Fig 5M). These findings indicate that SPARC stabilizes DOT1L through CBP-mediated acetylation. Although both P300 and CBP were upregulated by SPARC, P300 did not possess the ability to promote DOT1L acetylation (Fig. S5F). Furthermore, CBP knockdown in TGF-β1-induced HK-2 cells suppressed the elevated levels of fibrosis-related proteins, resembling the effects observed under hypoxic conditions induced by CoCl_2_ (Fig. 5N, O), and also alleviated the SPARC-induced changes in fibrosis markers, including the reduction in ZO-1 and E-cadherin expression and the increase in N-cadherin expression (Fig. S5G). These findings collectively suggest that CBP is a critical mediator in SPARC-dependent DOT1L stabilization, driving the progression of fibrosis in PTECs.

### SPARC regulates CBP function via the MEK-ERK signaling pathway

**Figure 6:**
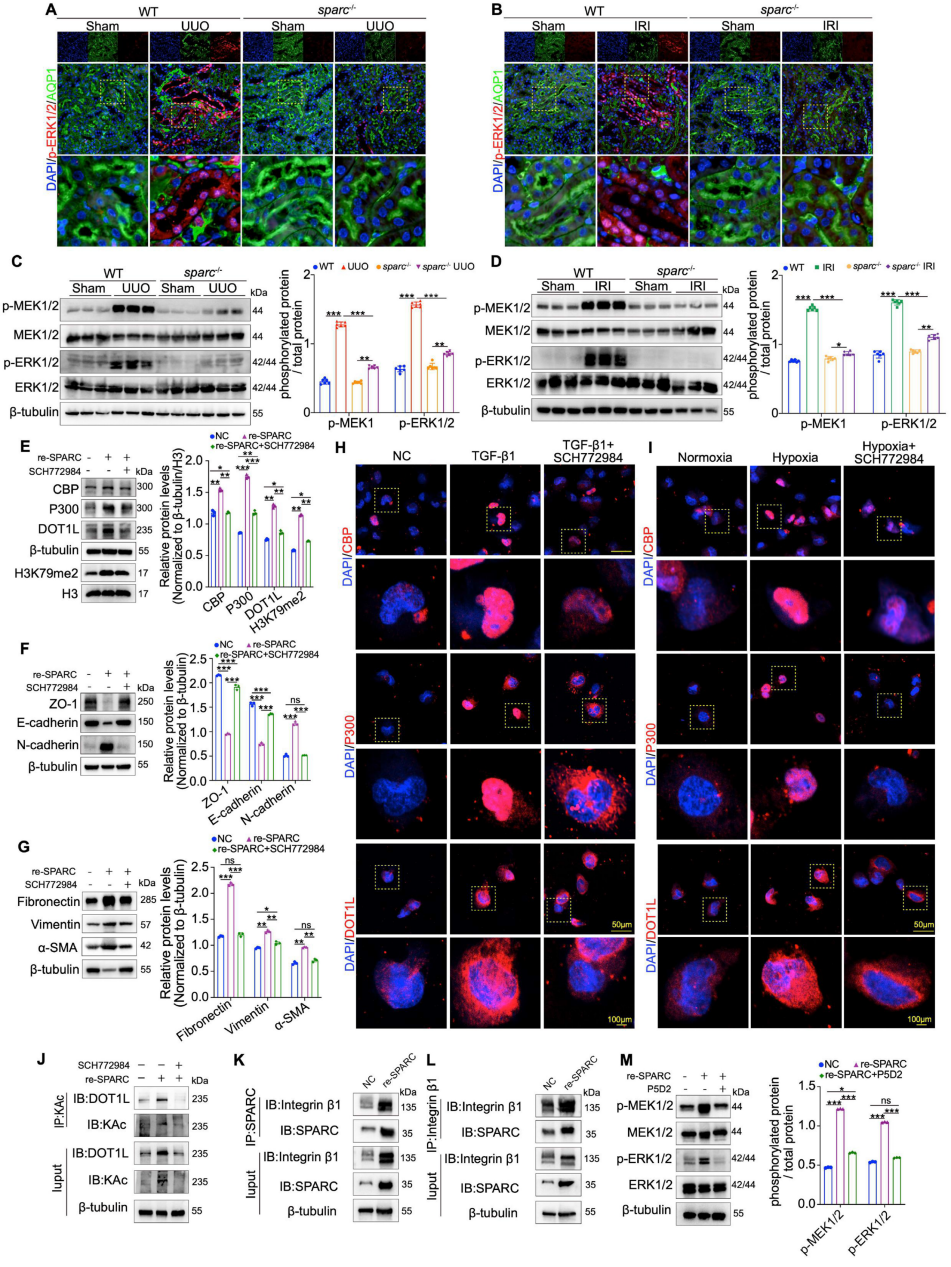
** Inhibition of the MEK/ERK pathway alleviates SPARC-induced TIF. A** and** B** Representative immunofluorescence images showing colocalization of p-ERK1/2 and AQP1 in kidney sections from WT and *sparc*^-/-^ mice after UUO and IRI. **C** and** D** Western blot and quantitative data showing the expression of p-MEK1/2, MEK1/2, p-ERK1/2 and ERK1/2 in the kidneys of WT and *sparc*^-/-^ mice after UUO and IRI (n=6). **E** and **G** Protein expression in HK-2 cells pretreated with the SCH772984 prior to incubation with the recombinant SPARC protein (n=3). **H** and** I** Representative immunofluorescence images showing CBP, P300, and DOT1L expression in HK-2 cells pretreated with SCH772984 before induction with TGF-β1 or CoCl_2_. **J** Immunoprecipitation with an anti-acetyl-lysine antibody followed by immunoblotting for DOT1L showed that SPARC-induced DOT1L acetylation was markedly attenuated when the MEK-ERK signaling pathway was blocked. **K** and **L** Co-IP assays showing that incubation with recombinant SPARC protein significantly enhances the physical interaction between SPARC and Integrin β1 in HK-2 cells. **M** Western blot analysis showing that recombinant SPARC treatment significantly increased the phosphorylation levels of MEK1/2 and ERK1/2, which was markedly inhibited by the Integrin β1 neutralizing antibody P5D2 (n=3). Data are presented as the mean ± SD. Statistical significance is indicated as *ns*, no significance, *P < 0.05, **P < 0.01, ***P < 0.001.

CBP plays a critical role in SPARC-dependent DOT1L stability and fibrosis progression, yet the mechanism by which SPARC regulates CBP expression remains unclear. Understanding this molecular link is crucial for elucidating the pathways that drive renal fibrosis. Previous studies have shown that SPARC, as well as its family member SPARCL1, could inhibit cancer cell proliferation and migration via the MEK/ERK signaling pathway, and ERK phosphorylation has been linked to the activation of CBP^31^-^33^. Based on this, we investigated whether the MEK/ERK pathway is involved in SPARC-mediated activation of CBP. Immunohistochemical, immunofluorescent, and Western blot analyses showed elevated MEK1/2 and ERK1/2 phosphorylation in PTECs from UUO and IRI fibrotic kidneys, which were significantly reduced upon *SPARC* knockout (Fig. 6A-D and Fig. S6A, B). *In vitro*, SPARC interference in TGF-β1-stimulated HK-2 cells suppressed MEK/ERK phosphorylation, with a similar pattern observed under CoCl_2_-induced hypoxia (Fig. S6C, D). Further analysis revealed that when recombinant SPARC protein was incubated with HK-2 cells in the presence of the ERK inhibitor SCH772984, the SPARC-induced elevation in phosphorylated MEK/ERK was suppressed (Fig. S6E). This was accompanied by reduced expression of CBP, P300, DOT1L and H3K79me2, along with recovery of ZO-1 and E-cadherin expression and suppression of N-cadherin, as well as decreased expression of Fibronectin, Vimentin, and α-SMA (Fig. 6E-G). Immunofluorescence analysis confirmed that ERK inhibition alleviated SPARC-induced increases in CBP, P300, DOT1L, Fibronectin, and α-SMA expression (Fig. 6H, I and Fig. S6F, G). In addition, ERK inhibition reduced CBP-mediated DOT1L acetylation (Fig. 6J).

Because SPARC is a secreted protein that initiates intracellular signaling by binding to membrane receptors, we tested whether SPARC interacts with Integrin β1, a known upstream activator of the MEK-ERK^34^,^35^. Co-immunoprecipitation assays confirmed that SPARC could bind Integrin β1, and this interaction was enhanced upon SPARC overexpression (Fig. 6K, L). Treatment with P5D2, a neutralizing antibody against Integrin β1, attenuated SPARC-induced MEK-ERK activation (Fig. 6M). Collectively, these findings demonstrate that SPARC regulates CBP via Integrin β1-mediated MEK-ERK signaling, enhancing DOT1L acetylation and promoting TIF progression.

## Discussion

**Figure 7:**
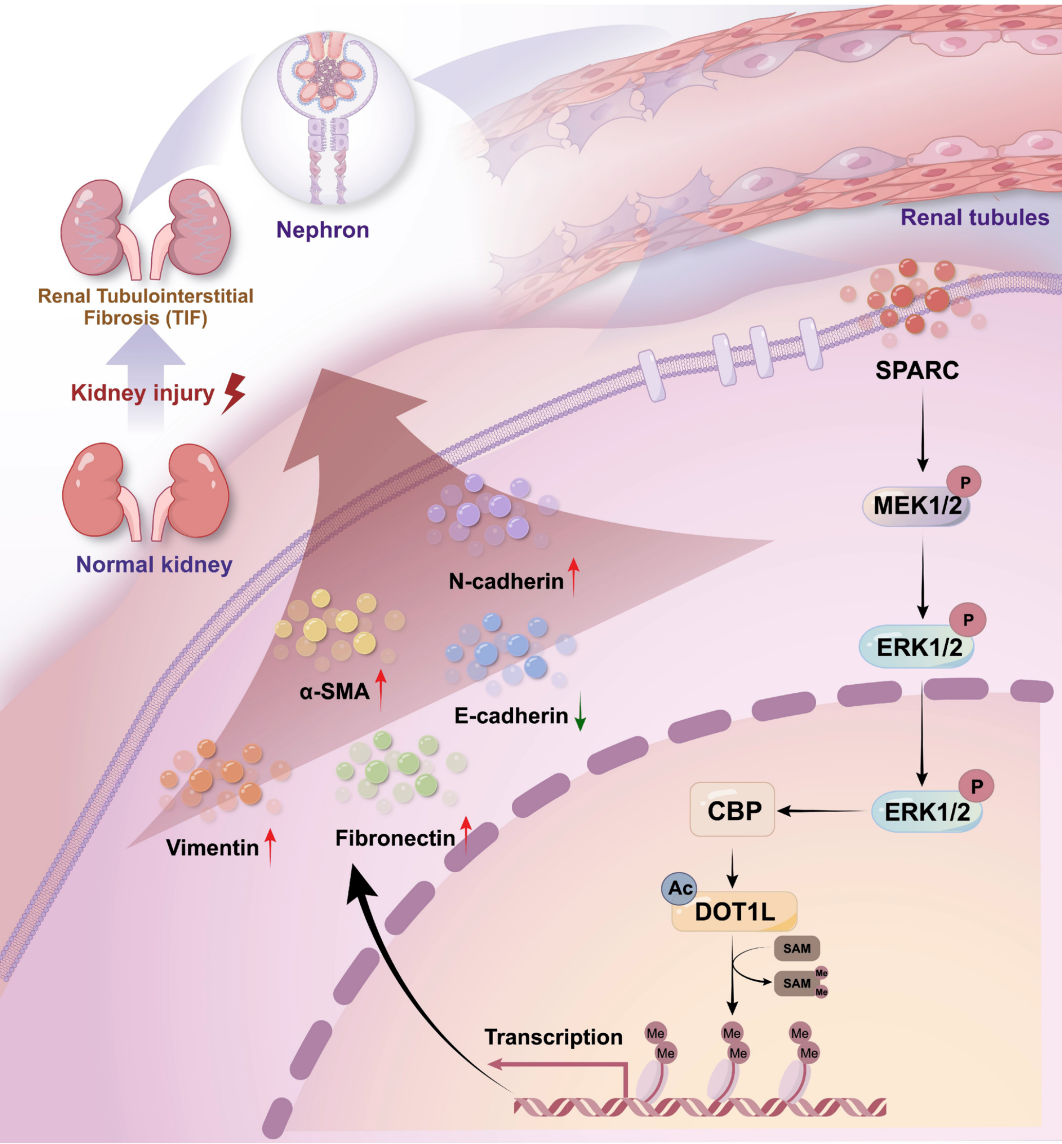
** Schematic diagram of the mechanism by which SPARC promotes TIF.** Diagram summarizing the proposed mechanism by which SPARC activates the CBP-DOT1L signaling axis via MEK/ERK pathway activation, promoting H3K79 methylation and renal interstitial fibrosis.

Renal fibrosis, particularly TIF, is a defining feature of progressive CKD and a major contributor to renal function decline worldwide^7^,^36^,^37^. Regardless of the underlying etiology, fibrosis leads to the replacement of functional renal tissue with scar tissue, which accelerates the deterioration of kidney function^38^,^39^. Despite significant progress has been made in understanding the molecular mechanisms driving fibrosis, substantial gaps remain. In this study, we identify SPARC as a novel mediator of TIF and demonstrate that elevated SPARC expression correlates with fibrosis severity. Mechanistically, SPARC regulates expression level of CBP via the MEK-ERK signaling pathway, stabilizes DOT1L and enhances H3K79me2 levels, collectively driving TIF progression (Fig. 7). Consistently, SPARC knockout markedly ameliorates renal fibrosis, suggesting that targeting the SPARC-mediated pathway may offer a promising therapeutic strategy for CKD.

Traditionally, SPARC is characterized as an ECM protein that, although secreted into the extracellular space, does not contribute structurally to the matrix. Instead, it regulates the interactions between cells and the ECM^40^,^41^. Previous studies have shown that SPARC, as a collagen-binding protein, plays a role in ECM assembly and TGF-β signaling^42^. Our findings reveal a distinct role for SPARC in renal fibrosis, whereby SPARC binds the membrane receptor Integrin β1, activating MEK-ERK signaling, and in turn regulates CBP. ERK phosphorylation-mediated upregulation of CBP protein levels is well-documented, and our study uncovers a novel mechanism by which SPARC regulates CBP via MEK-ERK, enhancing DOT1L stability^43^,^44^. This cascade drives fibrosis-related proteins expression in PTECs, thereby promoting TIF. Notably, we observed that SPARC is primarily localized in the cytoplasm of PTECs, and its presence in the culture supernatant confirms secretion, suggesting that the cytoplasmic signal reflects steady-state accumulation within transport vesicles.

DOT1L, a histone methyltransferase, is a known key epigenetic modifier in both lung and renal fibrosis. Inhibition of DOT1L has shown promise in alleviating fibrosis by blocking fibroblast activation and EMT^23^,^45^. In our study, SPARC enhanced DOT1L protein stability, whereas *SPARC* knockout reduced DOT1L levels and improved TIF. Further mechanistic analyses revealed that SPARC regulates CBP via the MEK-ERK pathway to acetylate DOT1L, which is consistent with previous studies that CBP-mediated acetylation enhances DOT1L stability and promotes cancer metastasis^30^. Although SPARC upregulated both CBP and its homolog P300, only CBP directly acetylated DOT1L, highlighting functional divergence. P300 may still contribute to fibrosis via alternative substrates or chromatin remodeling, which warrants further investigation.

Macrophages play a dual role in renal fibrosis. M1 macrophages drive early-stage injury through pro-inflammatory responses, whereas M2 macrophages promote late-stage fibrosis by enhancing extracellular matrix deposition. Bone marrow-derived macrophages can also transition into myofibroblasts, serving as a key immune-stromal hub in fibrosis^24^,^25^. In our study, F4/80 immunofluorescence staining showed no significant change in macrophage infiltration after SPARC knockout. Nevertheless, this does not rule out a regulatory role of SPARC in macrophage biology. Previous studies have shown that SPARC modulates macrophage polarization through the TLR4 signaling pathway, suggesting that total macrophage numbers alone may not reflect changes in M1/M2 polarization or paracrine function^46^. SPARC may additionally act directly on PTECs to drive renal fibrogenesis independently of immune cells. Meanwhile, studies have also suggested that SPARC can promote renal inflammation, whether SPARC indirectly promotes fibrosis through inflammatory regulation, and how it specifically affects macrophage functional phenotypes, remain important questions for future investigation^47^.

Certainly, this study has certain limitations. First, because SPARC is a secreted protein, PTEC-specific overexpression or knockout would not fully prevent systemic effects, and thus our current design using global knockout mice cannot completely isolate the contributions of PTEC-derived SPARC to renal injury. Second, we did not directly assess CBP phosphorylation or perform mutagenesis to confirm ERK-mediated CBP phosphorylation in our model, although ERK is known to phosphorylate CBP at multiple sites^43^,^44^,^48^. Future studies using phospho-specific antibodies or mass spectrometry to map ERK-dependent CBP phosphorylation sites in the context of SPARC-induced fibrosis will help elucidate the precise molecular mechanism. Third, while we focused on MEK-ERK as a key upstream regulator, other signaling molecules may also participate in SPARC-mediated regulation, warranting further investigation.

## Conclusions

In summary, our findings suggest that SPARC could contribute to TIF through modulation of the CBP-DOT1L pathway via MEK-ERK signaling. These observations point to a possible role for SPARC in CKD progression and offer new perspectives on pathways underlying renal fibrosis.

## Supplementary Material

Supplementary figures and tables.

## Data Availability

All data and materials used in this study are available from the corresponding author upon reasonable request.
