# Supplementary material for: SPARC Drives Tubulointerstitial Fibrosis through Regulating the CBP-DOT1L Pathway
Source: Int J Biol Sci. 2026 May 18;22(10):5495–508. doi: 10.7150/ijbs.133879 (PMC13215461; doi:10.7150/ijbs.133879)
Supplement: Supplementary file 1 — Supplementary figures and tables. [file ijbsv22p5495s1.pdf]

Supplemental Figure 1

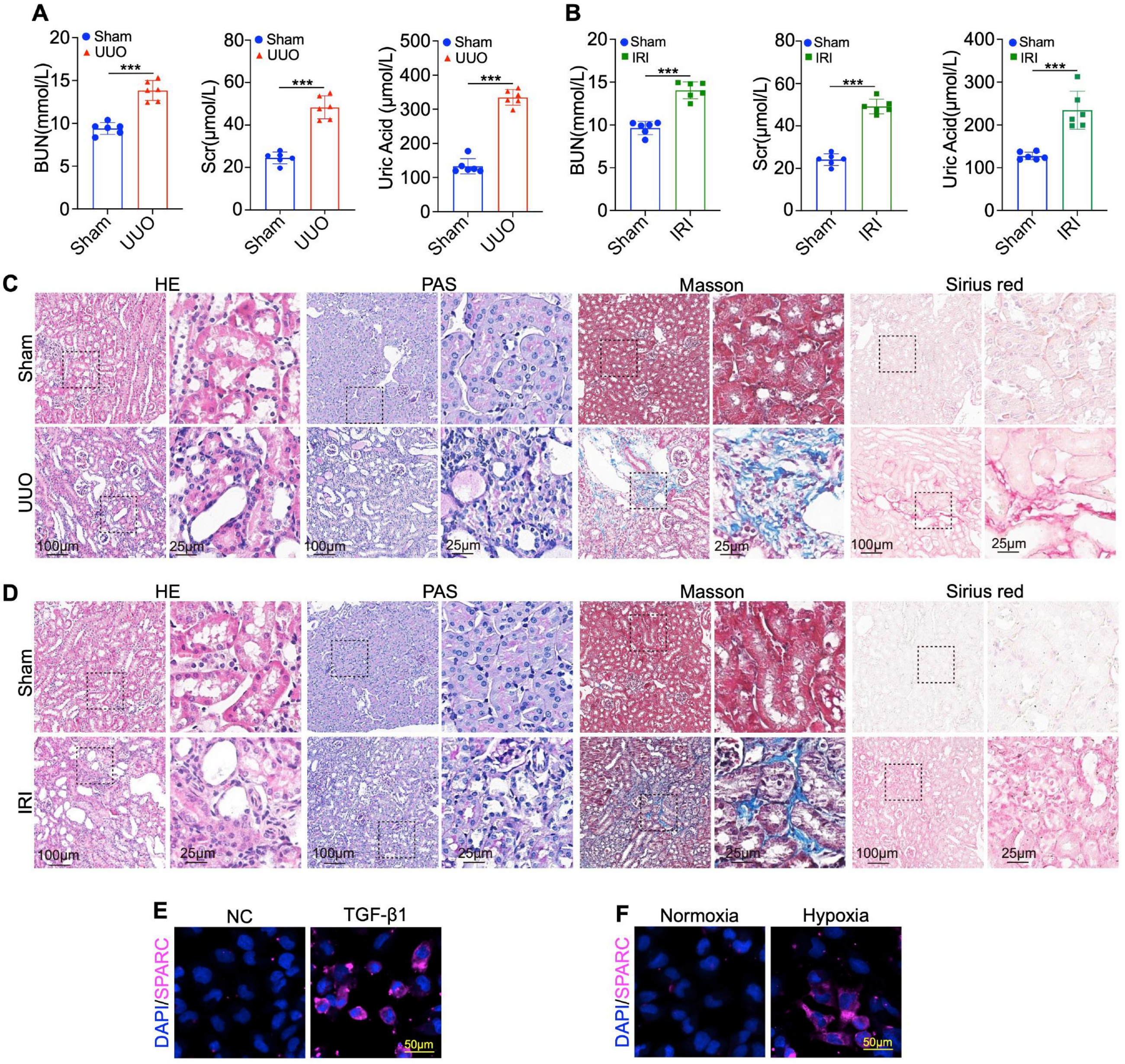

Supplementary Figure 2

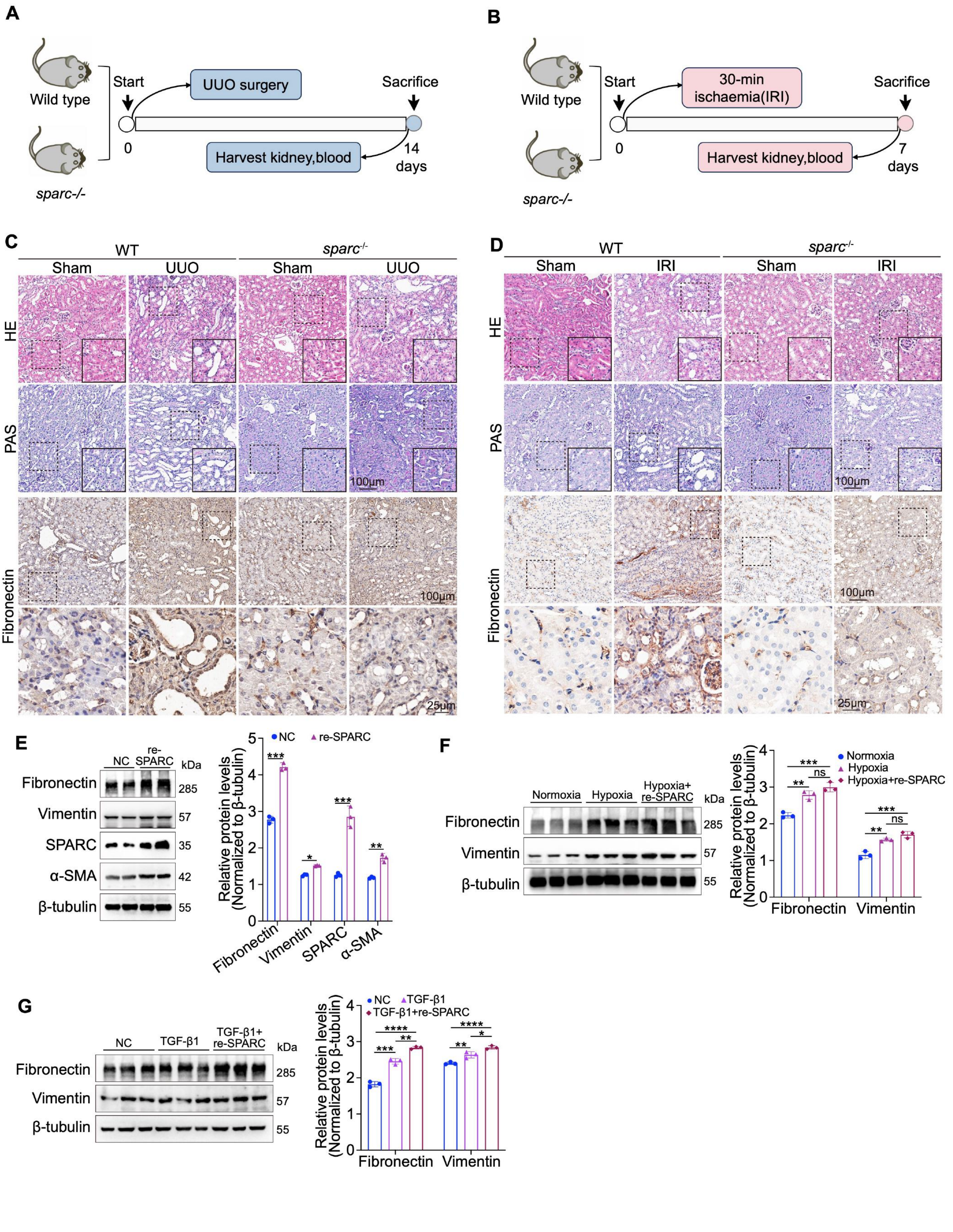

Supplemental Figure 3

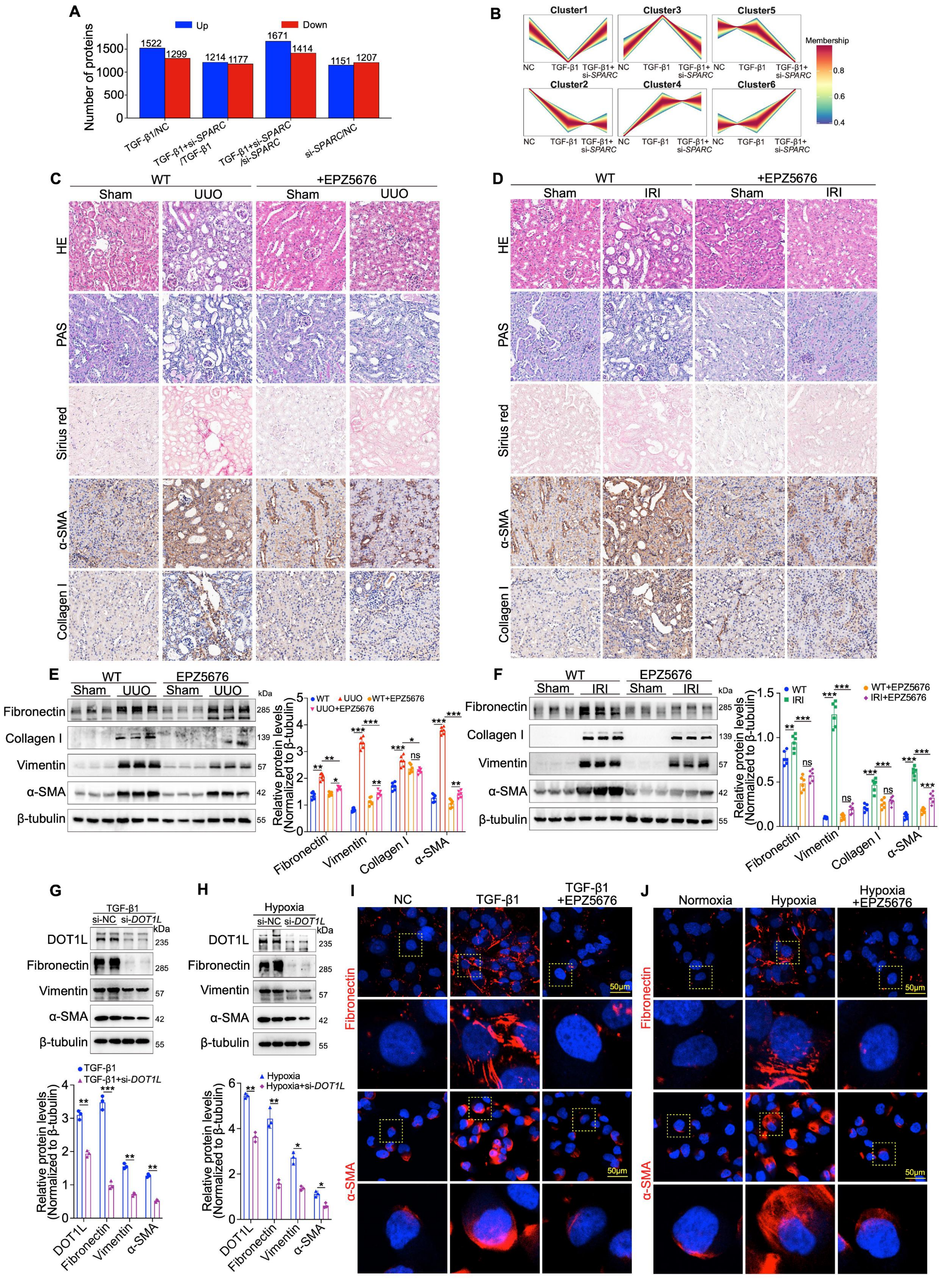

Supplemental Figure 4

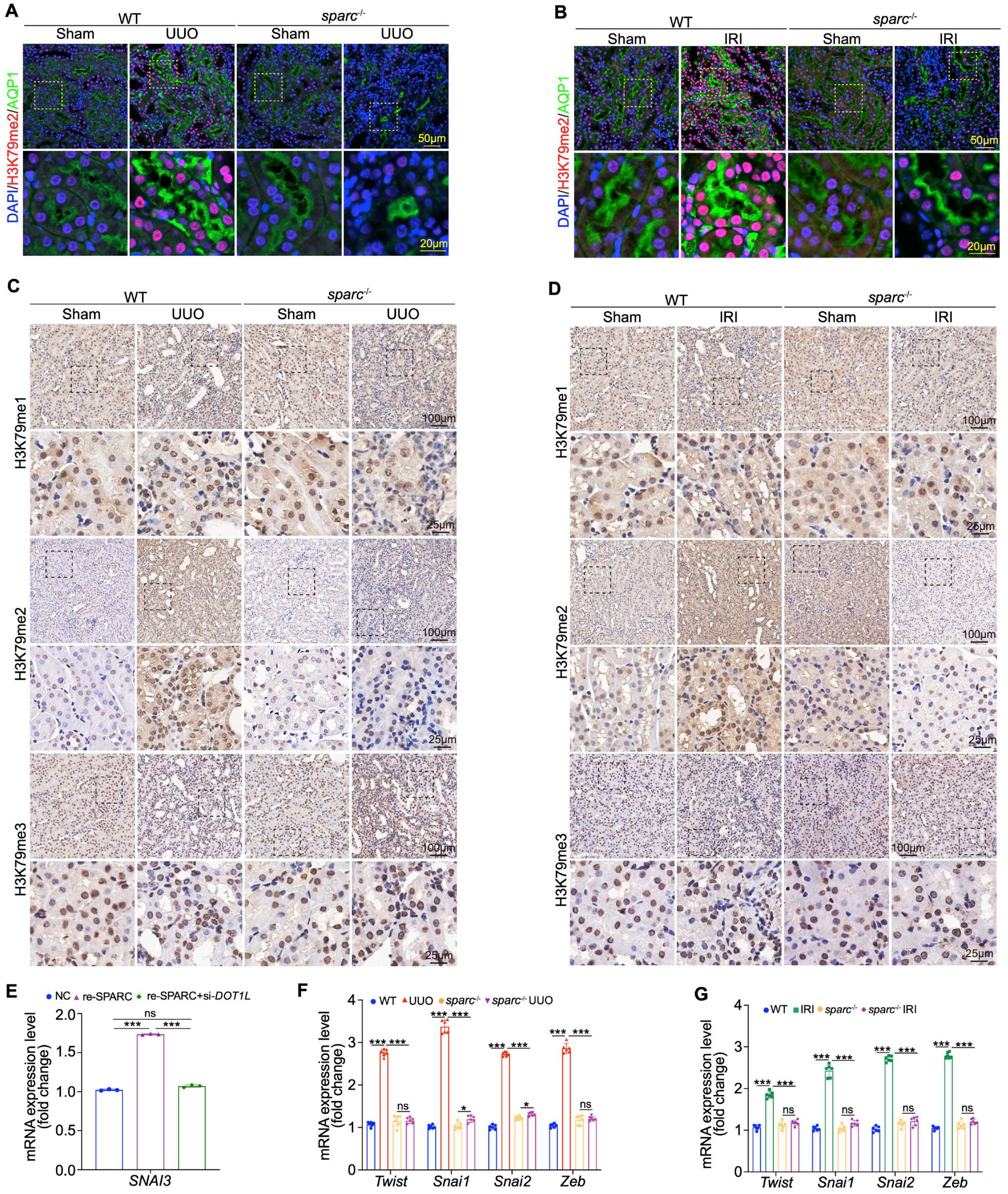

Supplemental Figure 5

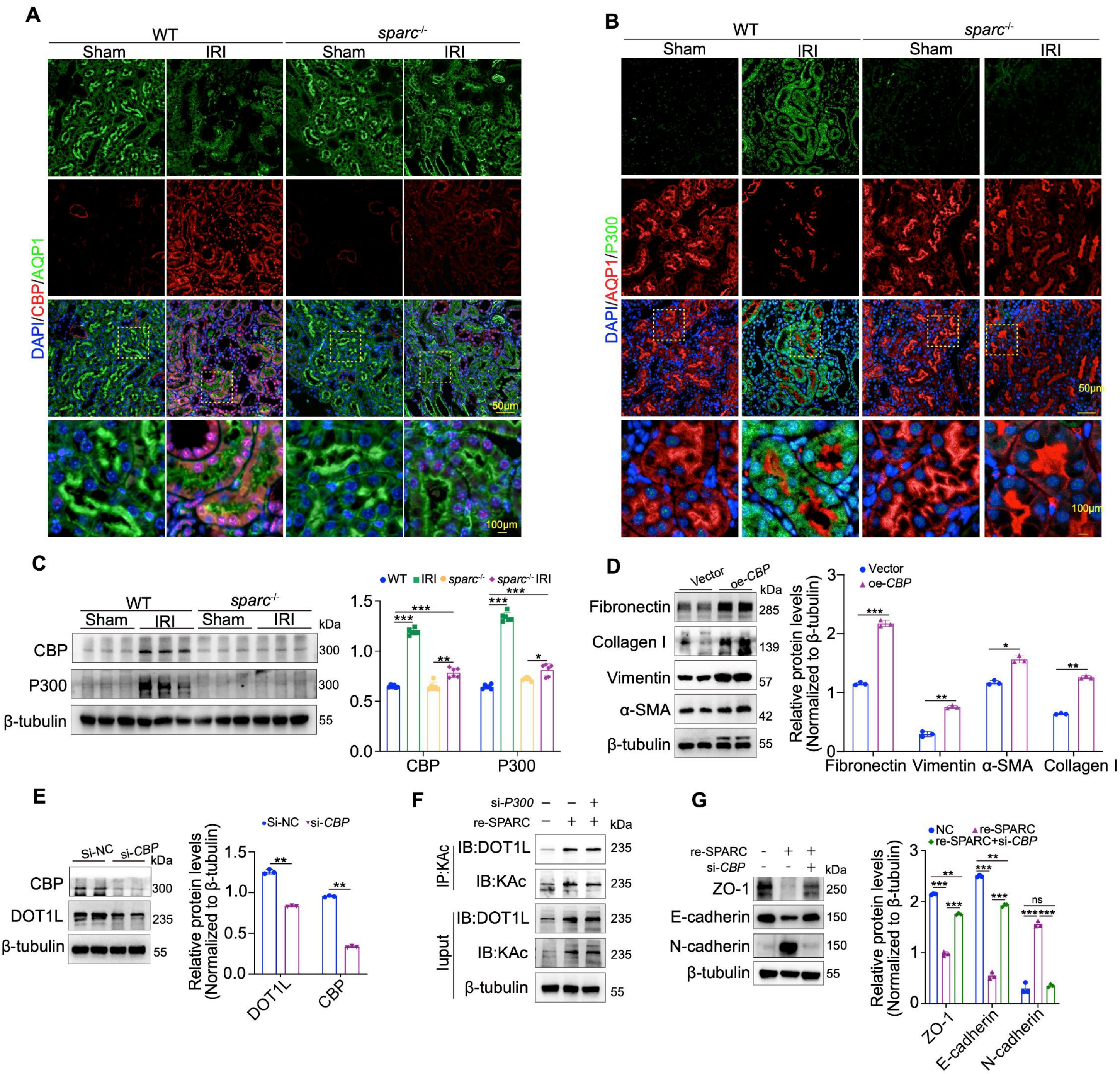

Supplemental Figure 6

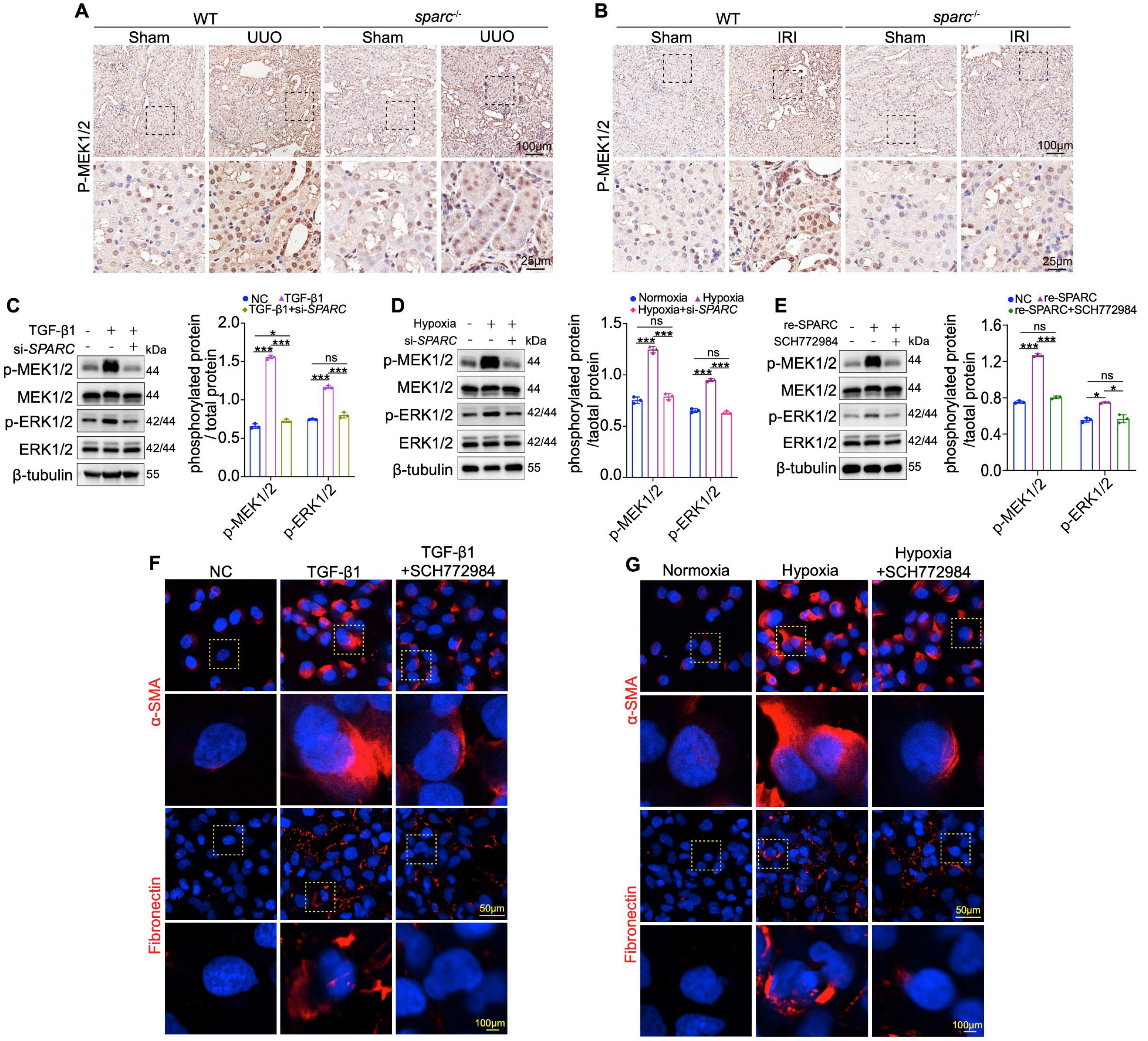

Supplemental Table 1. Clinical information of subjects

Group	Nonrenal fibrosis		Renal fibrosis		
Pathological diagnosis	HC	AKI	IgAN	IgAVN	DN
Genger (male, %)	4 (4/6)	3(3/6)	6 (6/10)	3(3/7)	5(5/8)
Age (year)	6.5±1.6	8.9±1.3	7.3±5.2	10.7±6.1	53.7±8.4
Scr (μmol/l)	64.3±24.8	121.3±34.9	110.8±35.7	131.2±28.4	128.4±25.6
BUN (mmol/l)	5.1±1.9	11.9±3.2	10.3±2.7	10.7±4.5	12.1±3.8
eGFR(ml/min/1.73m^2)	112.3±38.7	77.3±14.5	82.9±15.3	75.4±25.5	62.9±20.7

Results are expressed as mean ± SD. HC, healthy control; AKI, acute kidney injury; IgAN, IgA nephropathy; IgAVN, IgA vasculitis with nephritis; DN, diabetic nephropathy; Scr, serum creatinine; BUN, blood urea nitrogen; eGFR, estimated glomerular filtration rate.

Supplemental Table 2. Primer sequences for genotyping

Number	Sequence Forward	Sequence Reverse
1	TCTTGGAGTAAGACTGGAGCTGGG	GAAGAACCCACAGTAGACAATGGGC
2	CGGTCTCCCTTGGCATGAAAC	GCATGATGCTTCAGAAACCCAGA

Supplemental Table 3. List of Primary antibodies

Primary antibody	Source	Provider	Catalog	Application
AQP1	mouse	Santa Cruz	sc-55466	IF(1:50)
LTL	/	VectorLabs	FL-1321	IF(1:200)
F4/80	Rabbit	CST	70076S	IF(1:500)
SPARC	mouse	Proteintech	66426-1-Ig	IF (1:200)
CBP	Rabbit	Immunoway	YM8348	ICC/IF (1:200), WB (1:1000)
Acetyl-lysine	Rabbit	Abcam	ab190479	IP (1:400)
P300	Rabbit	Abclonal	A13016	ICC/IF (1:200)
P300	Rabbit	Proteintech	20695-1-AP	WB (1:1000)
DOT1L	Rabbit	Novas	NB100-40845	ICC/IF (1:200)
DOT1L	Rabbit	Abcam	ab239358	WB (1:1000)
DOT1L	Rabbit	Abcam	ab72454	IP (2-5 μg/mg of lysate)
α-SMA	Rabbit	Huabio	ET1607-53	ICC (1:400), IHC (1:400)
α-SMA	Mouse	Proteintech	67735-1-Ig	IF (1:100)
Collagne I	Rabbit	Immunoway	YT5769	IF (1:200), WB (1:1000)
H3K79me1	Rabbit	Huabio	HA722848	IHC (1:200)
H3K79me2	Rabbit	Abcam	ab177185	IF/IHC (1:200), WB (1:1000)
H3K79me3	Mouse	Immunoway	YM3091	IHC (1:200)
N-cadherin	Rabbit	Immunoway	YM8097	IHC (1:400), WB (1:1000)
E-cadherin	Rabbit	Proteintech	20874-1-AP	IHC (1:1000)
E-cadherin	Rabbit	Immunoway	YT1454	WB (1:1000)
p-ERK 1/2	Rabbit	Immunoway	YM8452	IF (1:200), WB (1:3000)
ERK1/2	Rabbit	Immunoway	YM8336	WB (1:3000)
p-MEK 1/2	Rabbit	Immunoway	YM8556	IHC (1:200), WB (1:3000)
MEK1/2	Rabbit	Immunoway	YM8273	WB (1:3000)
Fibronectin	Rabbit	Abcam	ab2413	ICC/IHC (1:200), WB (1:2000)
SPARC	Rabbit	Abcam	ab225716	WB (1:1000) for HK-2 cells
SPARC	Rabbit	Abcam	ab290636	WB (1:1000) for mice
β-tubulin	Mouse	Proteintech	66240-1-Ig	WB (1:3000)
GAPDH	Mouse	Huabio	EM1101	WB (1:2000)
Vimentin	Mouse	Santa Cruz	sc-373717	WB (1:500)
PLK3	Rabbit	Proteintech	10977-1-AP	WB (1:1000)
ZO-1	Mouse	Proteintech	66452-1-Ig	WB (1:1000)
H3	Rabbit	Selleck	F0057	WB (1:1000)
Intergrin β1	Rabbit	Abcam	ab183666	IP (1:400)

Supplemental Table 4. Primer sequences for siRNA

Gene Name	Sequence Forward	Sequence Reverse
si-*SPARC*	AGACAGAGGUGGUGGAAGATT	UCUUCCACCACCUCUGUCUTT
si-*DOT1L*	GCAAGAAGAAGCUAAACAA	UUGUUUAGCUUCUUCUUGC
si-*CBP*	GUUUCUCAAGGGAUGAAUU	AAUUCAUCCCUUGAGAAAC
si-*P300*	CGAGUCUUCUUUCUGACUCAA	UUGAGUCAGAAAGAAGACUCG

Supplemental Table 5. Primer sequences for qPCR

Gene Name	Sequence Forward	Sequence Reverse
Human-*DOT1L*	GAGACCTCCTTCGACCTGGT	CGACGCCATAGTGATGTTTGC
Human-*PLK3*	GGCACTGTCCAGGTGAACTT	CAAGCACTACGATTTCGGGC
Human-*SNAI3*	TCCTTCCTGGTGAAAACGCA	GCAGGCACCATTGATTTCTCT
Mouse-*Snai1*	CACACGCTGCCTTGTGTCT	GGTCAGCAAAAGCACGGTT
Mouse-*Snai2*	TGGTCAAGAAACATTTCAACGCC	GGTGAGGATCTCTGGTTTTGGTA
Mouse-*Zeb*	GCCTGCCACTCGGCTTTAT	CGGTACACGGTGAACTCGT
Mouse-*Twist*	CGCTACAGCAAGAAATCGAGC	GCTGAGCTTGTCAGAGGGG

Supplemental Table 6. Primer sequences for ChIP-qPCR

Gene Name	Sequence Forward	Sequence Reverse
Human-*SNAI3*	TCCTTCCTGGTGAAAACGCA	CGGGGATCGGGTAGTCAGTA

Supplemental materials and methods

Animals and treatment

Sparc-KO mice (C57BL/6 background, strain No. T015369) were generated using CRISPR/Cas9 technology and purchased from GemPharmatech (Nanjing, Jiangsu, China). Male wildtype C57BL/6 mice were used as controls. Genotype identification was performed by PCR using genomic DNA extracted from tail tissues, with primer sequences provided in Supplementary Table 2. Mice were housed in a specific pathogen-free environment at 23 ± 1°C under a 12-hour light/dark cycle with free access to food and water. Mice (8-12 weeks old) were subjected to IRI or UUO to fibrosis models. For the UUO model, a midline abdominal incision was made under anesthesia to expose the left ureter, which was ligated with 5-0 steel wire. The sham-operated group underwent similar procedures without ureteral ligation. In the IRI model, the left renal pedicle was clamped for 30 minutes at 37°C under anesthesia. The IRI mice were sacrificed 7 days after modeling, and the UUO mice were sacrificed 14 days after modeling. Urine, blood, and kidney samples from the operated side were collected for further analysis. To inhibit DOT1L, 35 mg/kg EPZ5676 (S7062, Selleck, USA), dissolved in 50 μL DMSO, was administered subcutaneously immediately following the surgical procedures. Daily injections of EPZ5676 continued for 6 consecutive days, with the control group receiving an equal volume of DMSO. All animal procedures were approved by the Ethics Committee of the Children's Hospital of Chongqing Medical University (IACUC approval No. CHCMU-IACUC20250804007).

Histopathological staining

Kidneys were fixed in 4% formaldehyde, embedded in paraffin, and sectioned at 3.5 μm. HE, PAS (G1120, Solarbio, China), Masson (DC0033, Leagene, China), and Picrosirius Red (DC0041, Leagene, China) staining were performed according to the manufacturer's instructions.

Immunofluorescence and immunohistochemistry

For immunofluorescence, kidney tissue slides were dewaxed, rehydrated, and subjected to heat-induced antigen retrieval in EDTA buffer (pH 9.0, ZLI-9072, ZSGB-BIO, China). Cells were fixed in 4% paraformaldehyde for 15 minutes, then blocked with goat serum for 1.5 hours at room temperature. Primary antibodies were incubated overnight at 4°C, followed by secondary antibody incubation at room temperature for 1 hour. Nuclei were counterstained with DAPI (C1002, Beyotime, China) for 10 minutes. Images were captured using a confocal microscope (Nikon A1R,

Japan). For immunohistochemistry, sections were incubated with an enhanced polymer detection kit (PV9001, ZSGB-BIO, China), primary antibodies overnight at 4°C, and developed with DAB substrate (DA1010, Solarbio, China). Information on all primary antibodies used were summarized in Supplementary Table 3.

Cell culture and treatment

Human Kidney-2 (HK-2, RRID: CVCL_0302) cells were obtained from Haixing Biosciences (Lot: TCH-C400) and cultured in HK-2-specific medium (TCH-G400, Haixing Biosciences) at 37°C with 5% CO_2. The cells were confirmed to be free of Mycoplasma contamination. To induce fibrosis, HK-2 cells were starved for 12 hours and then treated with 10 ng/mL TGF-β1 (100-21, PeproTech, USA) or 700 μM cobalt dichloride ($CoCl_2$) (S9490, Selleck, USA) for 24 hours. For SPARC stimulation, cells were incubated with recombinant SPARC protein (HY-P71094, MCE, USA) at 0.5 μg/mL for 48 hours. DOT1L inhibition was achieved by pre-treating cells with 50 μM EPZ5676 (S7062, Selleck, USA) for 1 hour before recombinant SPARC protein treatment. To inhibit the MEK-ERK pathway, cells were treated with SCH772984 (S7101, Selleck, USA) at 5 μM. To neutralize integrin β1, cells were treated with anti-integrin β1 neutralizing antibody (clone P5D2, MAB1959Z, Merck Millipore, USA) at 10 μg/mL. HK-2 cells were transfected with *SPARC*, *DOT1L*, *CREBBP* or *P300* siRNAs (GENE CREATE, Wuhan, China) or scrambled siRNA (negative control) using jetPRIME transfection reagent (Polyplus-transfection, Illkirch, France). Specific information on siRNA sequences were provided in Supplementary Table 4. Gene silencing efficiency was confirmed by Western blot, with a knockdown efficiency of >70% considered successful.

Western blot analysis

Proteins were extracted from kidney tissues or cells using RIPA buffer (WB3100, NCM Biotech, China) with protease inhibitors (C0001, TargetMol, USA). Protein concentration was determined using a BCA kit (MA0082, Meilunbio, China). Proteins were separated by SDS-PAGE and transferred to PVDF membranes, which were blocked with 5% non-fat milk for 1 hour at room temperature. Membranes were incubated with primary antibodies overnight at 4°C, followed by HRP-conjugated secondary antibodies (1:10000, ZSGB-BIO, China). Blots were developed using ECL substrate (34580, ThermoFisher Scientific, USA) and quantified with Image J software.

ELISA

The concentration of SPARC in cell culture supernatants was measured using a commercial ELISA kit (JL13065-96T, Jianglai Bio, China) according to the manufacturer's protocol. Briefly, samples collected from NC, TGF-β1, Normoxia, and Hypoxia groups were added to pre-coated 96-well plates and incubated with detection antibody, followed by HRP-conjugated streptavidin. After adding TMB substrate, the absorbance was read at 450 nm.

Quantitative PCR (qPCR)

Total RNA was isolated from kidney tissues or HK-2 cells using the SteadyPure Kit (AG21023, Accurate Biology, China). cDNA synthesis was performed using the HiScript IV First Strand Kit (R412-02, Vazyme, China). qPCR was performed with Taq Pro Universal SYBR qPCR Master Mix (Q712-03, Vazyme), and relative mRNA expression was calculated using the $2^{-}\Delta\Delta Ct$ method. Primer information is provided in Supplementary Table 5.

CUT&Tag Assay

CUT&Tag was performed using the TD904 Kit (Vazyme, China) following the manufacturer's instructions. Briefly, cells were collected, washed, and bound to pre-activated ConA Beads Pro, then incubated overnight at 4°C with H3K79me2 (Abcam, ab177185, USA) antibody, and corresponding secondary antibody (Ab207, Vazyme, USA), prior to treatment with hyperactive PG-TN5 transposon for targeted chromatin fragmentation. Fragmented DNA was then purified and amplified via polymerase chain reaction (PCR). High-throughput sequencing was performed on the Illumina NovaSeq platform. Raw sequencing data were analyzed by All Bioknow Biotechnology Co., Ltd (Chongqing, China).

ChIP-qPCR

Chromatin immunoprecipitation (ChIP) was performed using the Chromatin Immunoprecipitation Kit (Beyotime, P2080S, China). Precleared chromatin was incubated overnight at 4°C with either anti-H3K79me2 antibody (Abcam, ab177185, USA) or normal rabbit IgG (Beyotime, A7016, China), serving as a negative control. After purified using the kit (Beyotime, D0033, China), the immunoprecipitated DNA was analyzed by quantitative real-time PCR (qPCR) using primers specific for the *SNAI3* promoter region, as detailed in Supplementary Table 6. Enrichment of immunoprecipitated DNA was calculated as a percentage of input DNA (% Input) and normalized to the IgG control.

Cycloheximide (CHX) Chase Assay

Supplementary Fig.1 Assessment of renal function and histopathological changes in UUO and IRI mice. **A** and **B** Serum levels of creatinine (Scr), blood urea nitrogen (BUN), and uric acid in mice after UUO or IRI surgery. **C** and **D** Representative kidney sections showing HE, PAS, Masson, and Sirius red staining in sham-operated and UUO or IRI groups. **E** and **F** Representative immunofluorescence images showing SPARC expression in HK-2 cells treated with TGF-β1 and $CoCl_2$. Data are presented as the mean ± SD. Statistical significance is indicated as *ns*, no significant, **P* < 0.05, ***P* < 0.01, ****P* < 0.001.

Supplementary Fig. 2 Interfering with SPARC expression alleviates TIF. **A** and **B** Experimental design. Kidneys and blood were collected from WT and *sparc*$^{-/-}$ mice after UUO or IRI surgery. **C** and **D** Representative images of HE, PAS and immunohistochemical of Fibronectin staining of WT and *sparc*$^{-/-}$ mouse kidneys after UUO and IRI. **E** Western blot showing the expression of Fibronectin, Vimentin and α-SMA in HK-2 cells incubated with 0.5 μg/ml recombinant SPARC protein for 48 hours (n=3). **F** Western blot showing the expression of Fibronectin and Vimentin in HK-2 cells co-incubated with $CoCl_2$ and recombinant SPARC protein. **G** Western blot showing the expression of Fibronectin and Vimentin in HK-2 cells co-incubated with TGF-β1 and recombinant SPARC protein (n=3). Data are presented as the mean ± SD. Statistical significance is indicated as *ns*, no significance, **P* < 0.05, ***P* < 0.01, ****P* < 0.001.

Supplementary Fig. 3 Inhibiting DOT1L can ameliorate TIF. **A** Bar chart showing the statistics of the number of upregulated and downregulated proteins between two groups. **B** The line chart showing changes in protein expression levels among different groups in each expression pattern cluster. One line represents one protein, and the color of the line indicates the membership degree of the protein in the current class. **C** and **D** From top to bottom are the renal HE, PAS, Sirius red, α-SMA and Collagen I stain of WT mice injected or not injected with EPZ5676 after UUO and IRI. **E** and **F** Western blot and quantitative data showing the expression of Fibronectin, Collagen I, Vimentin and α-SMA in the kidneys of WT mice injected or not injected with EPZ5676 after UUO and IRI (n=6). **G** and **H** Western blot and quantitative data showing the expression of DOT1L, Fibronectin, Vimentin and α-SMA in HK-2 cells which treated with TGF-β1 or $CoCl_2$ following DOT1L knockdown (n=3). **I** and **J** Immunofluorescence showing Fibronectin and α-SMA

expression in HK-2 cells pretreated with EPZ5676 1 hour prior to TGF-β1 or $CoCl_2$ stimulation. Statistical significance is indicated as *ns*, no significance, **P* < 0.05, ***P* < 0.01, ****P* < 0.001.

Supplementary Fig. 4 Knockout of *SPARC* could reduce the level of H3K79 di-methylation and decrease the expression of fibrosis-related transcription factors. A and **B** Immunofluorescence images showing colocalization of H3K79me2 with AQP1 in kidney tissues from WT and *sparc*$^{-/-}$ mice after UUO and IRI. **C** and **D** Representative immunohistochemical staining of H3K79me1, H3K79me2 and H3K79me3 in kidney sections from WT and *sparc*$^{-/-}$ mice after UUO and IRI. **E** Relative *SNAI3* mRNA expression in HK-2 cells determined by qPCR (n=3). **F** and **G** qPCR analysis of *Twist*, *Snai1*, *Snai2* and *Ze*b mRNA expression in kidney tissues of WT and *sparc*$^{-/-}$ mice UUO and IRI (n=6). Data are presented as the mean ± SD. Statistical significance is indicated as *ns*, no significance, **P* < 0.05, ***P* < 0.01, ****P* < 0.001.

Supplementary Fig. 5 SPARC enhances DOT1L protein stability via CBP and contributes to TIF. A and **B** Representative immunofluorescence images showing colocalization of CBP and P300 with AQP1 in kidney sections from WT and *sparc*$^{-/-}$ mice after IRI. **C** Western blot and quantitative data showing the expression of CBP and P300 in the kidneys of WT and *sparc*$^{-/-}$ mice after IRI (n=6). **D** Western blot and quantitative data showing the expression of Fibronectin, Collagen I, Vimentin and α-SMA in HK-2 cells transfected with CBP overexpression plasmid (n=3). **E** Western blot and quantitative data showing the expression of DOT1L in HK-2 cells with *CBP* knockdown. **F** Immunoprecipitation with an anti-acetyl-lysine antibody followed by immunoblotting for DOT1L showed that SPARC-induced DOT1L acetylation was not attenuated when P300 was knocked down. **G** Western blot analysis of ZO-1, E-cadherin and N-cadherin in HK-2 cells incubated with SPARC recombinant protein following *CBP* knockdown (n=3). Data are presented as the mean ± SD. Statistical significance is indicated as ns, no significance, **P* < 0.05, ***P* < 0.01, ****P* < 0.001.

Supplementary Fig. 6 SPARC activates the MEK/ERK pathway and contributes to TIF progression. A and **B** Immunohistochemical staining of p-MEK1/2 in WT and *sparc*$^{-/-}$ kidney sections after UUO and IRI. **C** and **D** Western blot and quantitative data showing expression of p-MEK1/2, MEK1/2, p-ERK1/2 and ERK1/2 in HK-2 cells which treated with TGF-β1 or $CoCl_2$ following *SPARC* knockdown (n=3). **E** Expression of p-MEK1/2, MEK1/2, p-ERK1/2 and ERK1/2 in HK-2 cells pretreated with the ERK inhibitor (SCH772984) 1 hour prior to incubation with the

recombinant SPARC protein (n=3). **F** and **G** Immunofluorescence showing Fibronectin and α-SMA expression in HK-2 cells pretreated with 5 μM SCH772984 before TGF-β1 or $CoCl_2$ stimulation. Data are presented as the mean ± SD. Statistical significance is indicated as ns, no significance, *$P < 0.05$, **$P < 0.01$, ***$P < 0.001$.

For the protein stability assay, HK-2 cells treated with or without recombinant SPARC protein, or co-treated with recombinant SPARC and si-*CBP*, were incubated with cycloheximide (CHX, 20 μg/mL) to block new protein synthesis. Cells were harvested at 0, 2, 4, 6, and 8 hours after CHX treatment, and total protein was extracted. The expression of DOT1L was detected by Western blot, with β-tubulin as an internal reference. The relative protein levels of DOT1L were quantified and normalized to the 0-hour time point to assess protein half-life and degradation rate.
